# Giant energy-storage density with ultrahigh efficiency in lead-free relaxors via high-entropy design

**DOI:** 10.1038/s41467-022-30821-7

**Published:** 2022-06-02

**Authors:** Liang Chen, Shiqing Deng, Hui Liu, Jie Wu, He Qi, Jun Chen

**Affiliations:** 1 Beijing Advanced Innovation Center for Materials Genome Engineering, University of Science and Technology Beijing, 100083 Beijing, China; 2 Department of Physical Chemistry, University of Science and Technology Beijing, 100083 Beijing, China; 3 School of Mathematics and Physics, University of Science and Technology Beijing, 100083 Beijing, China

**Keywords:** Ferroelectrics and multiferroics, Ceramics, Materials for energy and catalysis, Supercapacitors

## Abstract

Next-generation advanced high/pulsed power capacitors rely heavily on dielectric ceramics with high energy storage performance. However, thus far, the huge challenge of realizing ultrahigh recoverable energy storage density (*W*_rec_) accompanied by ultrahigh efficiency (*η*) still existed and has become a key bottleneck restricting the development of dielectric materials in cutting-edge energy storage applications. Here, we propose a high-entropy strategy to design “local polymorphic distortion” including rhombohedral-orthorhombic-tetragonal-cubic multiphase nanoclusters and random oxygen octahedral tilt, resulting in ultrasmall polar nanoregions, an enhanced breakdown electric field, and delayed polarization saturation. A giant *W*_rec_ ~10.06 J cm^−3^ is realized in lead-free relaxor ferroelectrics, especially with an ultrahigh *η* ~90.8%, showing breakthrough progress in the comprehensive energy storage performance for lead-free bulk ceramics. This work opens up an effective avenue to design dielectric materials with ultrahigh comprehensive energy storage performance to meet the demanding requirements of advanced energy storage applications.

## Abstract (web summary)

Dielectric ceramics are widely used in advanced high/pulsed power capacitors. Here, the authors propose a high-entropy strategy to design “local polymorphic distortion” in lead-free ceramics, achieving high energy storage performance.

## Introduction

Dielectric capacitors, as the core component of high/pulsed power electronic devices, are widely used in numerous fields such as hybrid electrical vehicles, microwave communications and distributed power systems^1–3^. This is because of the high-power density and ultrafast charge/discharge rates in dielectric capacitors, which store energy through the displacement of bound charged elements rather than chemical reactions as in batteries and solid oxide fuel cells^4,5^. However, the low recoverable energy storage density (*W*_rec_ generally <4 J cm^−3^) greatly limits the application fields of ceramic capacitors and their development toward device miniaturization and intelligence. Together with environmental protection, the design of high-performance lead-free energy storage capacitors has enormous potential in the global market.

A breakthrough in *W*_rec_ to 4 J cm^−3^ was realized in AgNbO_3_ (AN)-based ceramics by controlling the field-driven antiferroelectric-to-ferroelectric phase transition behavior^6^. Further breakthroughs in energy storage properties were also achieved in other representative lead-free ceramic systems, such as the excellent *W*_rec_ values of 7.4, 8.2, and 12.2 J cm^−3^ in (K,Na)NbO_3_ (KNN), BiFeO_3_ (BF), and NaNbO_3_ (NN)-based systems, respectively^7–9^. However, their poor energy storage efficiency (*η*) below 80% leads to high loss and heat generation after multiple runs, which causes the capacitors to undergo thermal breakdown and fail to work normally. Improving *η* and reducing heat generation can further increase the service life of the devices and save costs. Especially, in the context of energy saving and emission reduction, achieving high *η* on the basis of ultrahigh *W*_rec_ is necessary and significant, although there are great challenges^10,11^. To realize a super high *η*, numerous strategies, such as nanodomain/domain engineering^3,12^, superparaelectric state^1,13^, defect engineering^14,15^, enhanced antiferroelectric phase^6,16^, and enhanced local random field^17,18^, have been proposed to break the long-range ferroelectric order or decrease the remnant polarization (*P*_r_). In addition to domain structure adjustment, electric field control, such as narrowing the gap between *E*_F_ and *E*_A_, has also become an important strategy to improve efficiency in antiferroelectrics or relaxor antiferroelectrics. Recently, high *W*_rec_ and high *η* have been reported in some Bi_0.5_Na_0.5_TiO_3_ (BNT)-based lead-free ceramics^19–21^. However, the great challenge of realizing ultrahigh energy storage density (*W*_rec_ ≥10 J cm^−3^) with simultaneous ultrahigh efficiency (*η* ≥ 90%) still exists in lead-free ceramics and has not been overcome.

According to the theory of electrostatic energy storage, high-performance capacitors should have a large breakdown electric field *E*_b_, large Δ*P* (*P*_max_ − *P*_r_), delayed polarization saturation and a temperature/frequency-insensitive dielectric response. To realize these electrical factors at the same time, perovskites should be designed to show the following structural features: dense microstructure, fine grains, large polarizability under an electric field, fast response back to the nonpolar state, large barrier such as a local random field delaying the formation of a textured ferroelectric state, highly dynamic structure with a hysteresis-free response at the test frequency and temperature-insensitive structure. Therefore, breaking through the bottleneck of energy storage capacitors is a great challenge.

**Fig. 1:**
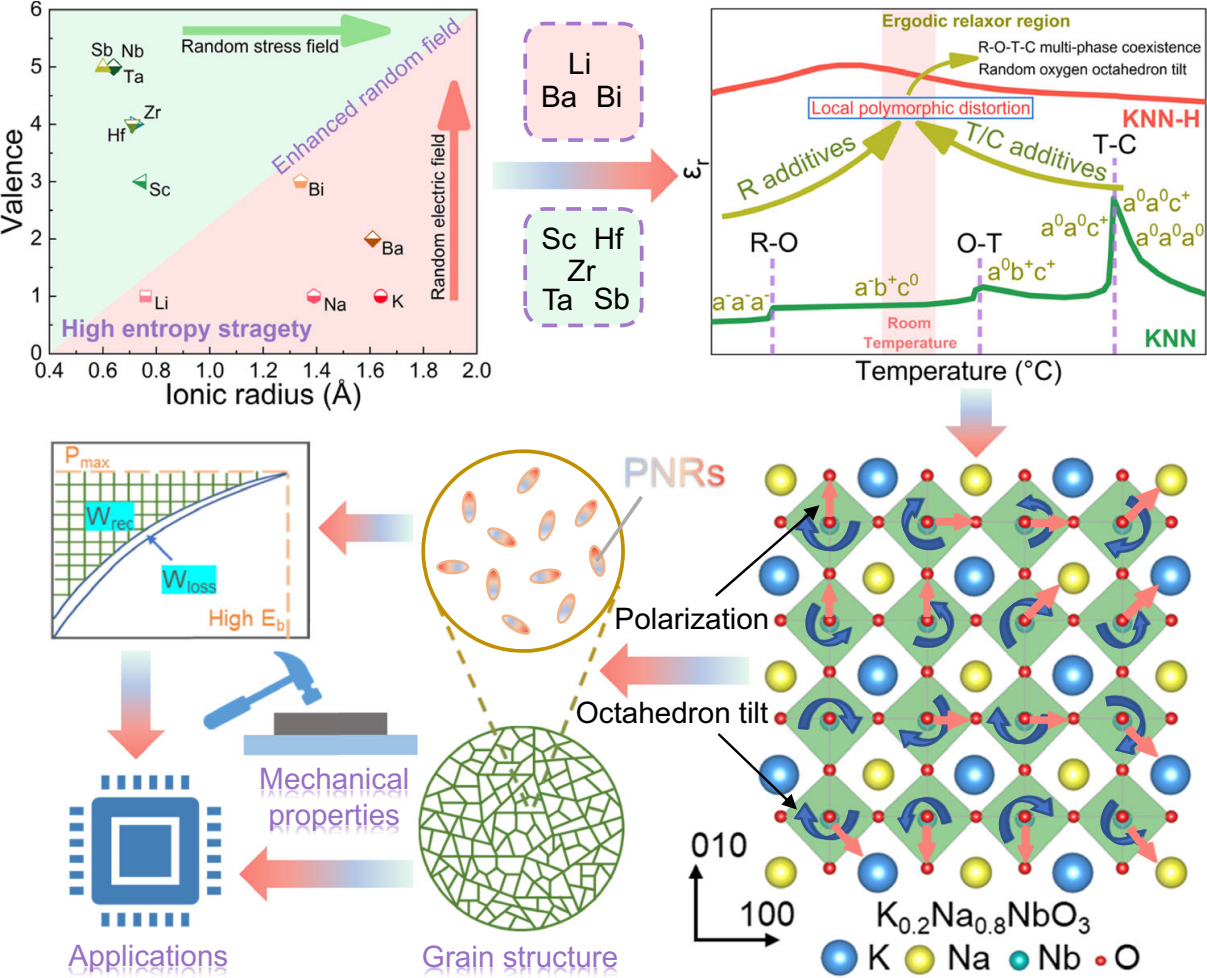
Schematic diagram of high-entropy design strategy for local polymorphic distortion and giant energy storage performance.

In this work, an effective high-entropy strategy is proposed to design “local polymorphic distortion” to enhance the comprehensive energy storage performance to break the status quo, which has usually been used for alloys^22,23^, oxides^24,25^, and metal carbides^26^ to improve mechanical properties. As shown in Fig. 1, numerous ions (Li^+^, Ba^2+^, Bi^3+^, Sc^3+^, Hf^4+^, Zr^4+^, Ta^5+^, Sb^5+^) with different ionic radii and valence states are introduced into K_0.2_Na_0.8_NbO_3_ lattices to enhance the random field, including the stress and electric field simultaneously. According to the phase boundary regulation of KNN-based ceramics by previous studies^27–31^, these ions are also considered as additives used to tailor *T*_R-O_, *T*_O-T_ and *T*_T-C_ to form rhombohedral-orthorhombic-tetragonal-cubic (R-O-T-C) multiphase nanoclusters coexisting at the local scale. Furthermore, different types of oxygen octahedral distortions exist in different nanophases, which would introduce randomly distributed oxygen octahedral tilt, further delaying polarization saturation. The local polymorphic distortion can effectively reduce the size of polar nanoregions (PNRs) and further decrease the loss when working under a strong electric field, providing great potential to improve *η* and *W*_rec_ at the same time. The mechanical performance could also be optimized by the high-entropy strategy to meet the requirements of practical applications. Encouragingly, a giant *W*_rec_ ~10.06 J cm^−3^ with an ultrahigh *η* ~90.8% is realized in lead-free relaxors, which is the optimal comprehensive energy storage performance reported to date for lead-free bulk ceramics. This proves that the high-entropy strategy can be used as a guide to develop new available energy storage materials with ultrahigh comprehensive properties.

## Results

### R-O-T-C multiphase nanoclusters coexistence

After the high-entropy strategy and preparation process optimization, movement of the polymorphic phase transition temperature, introduction of dielectric relaxation behavior and decrease of the dielectric loss can be detected at the same time, as shown in Supplementary Fig. 1. In addition, refined grains and compact microstructures with few pores and dense grain boundary structures can also be found according to the scanning electron microscopy (SEM) and transmission electron microscopy (TEM) results for the KNN-H ceramic in Supplementary Figs. 2 and 3. Owing to the strong dielectric relaxation behavior, PNRs revealed as a weak contrast or Moiré fringe structure^32^ can be observed by HR-TEM along [100]_c_, [110]_c_, and [111]_c_ due to the insufficient resolution.

**Fig. 2:**
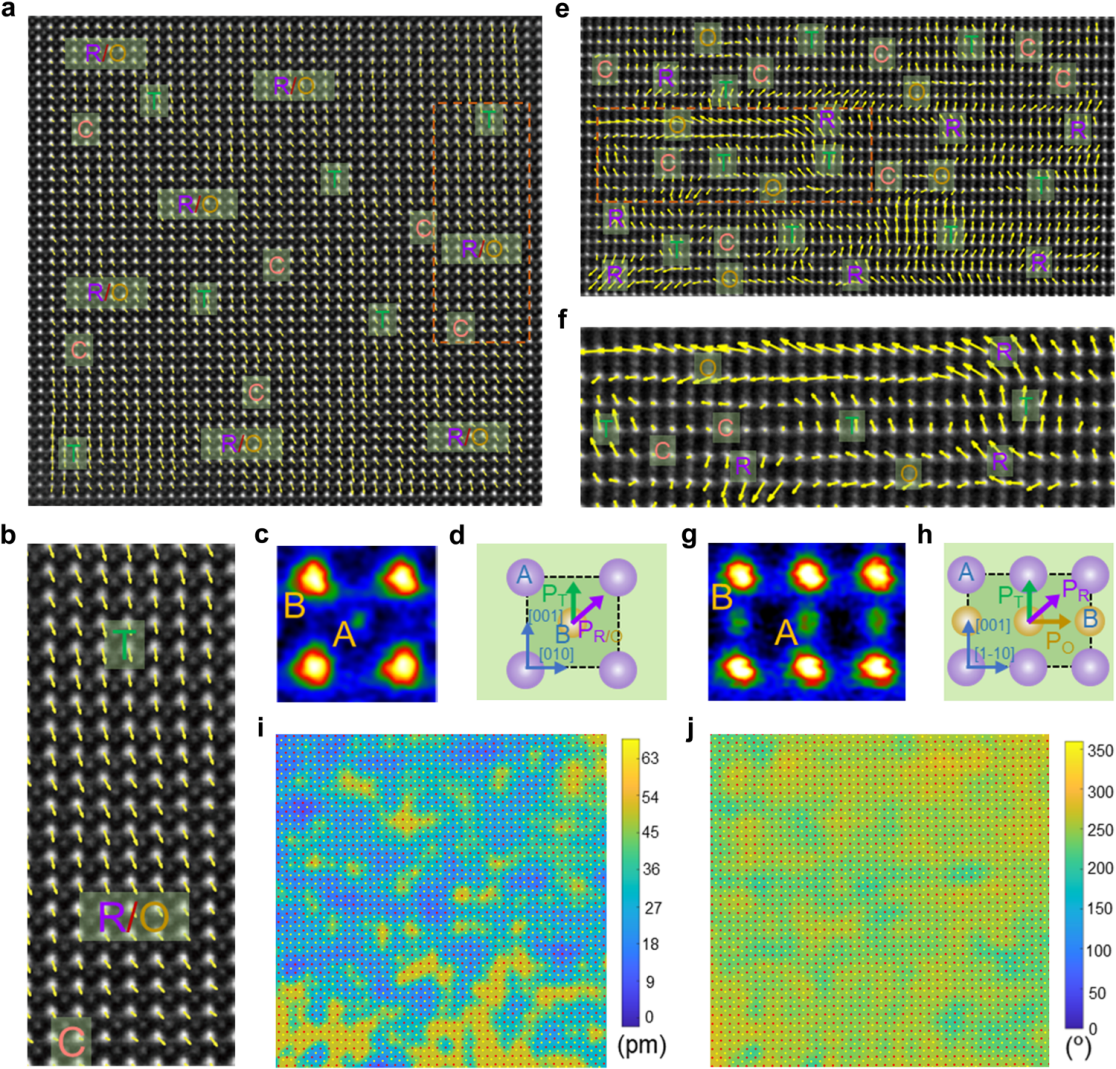
R-O-T-C multiphase nanoclusters coexistence. **a** Atomic-resolution HAADF STEM polarization vector image along [100]_c_. **b** Enlarged image of the marked area (dark red rectangle) in **a** showing the transition of polarization vectors from T to R/O to C. **c** Magnified image and **d** schematic projection of the unit cell along [100]_c_. **e** Atomic resolution HAADF STEM polarization vector image along [110]_c_. **f** Enlarged image of the marked area (dark red rectangle) in **e** showing the transition of polarization vectors from T to R to O to C. **g** Magnified image and **h** schematic projection of the unit cell along [110]_c_. **i** Polarization magnitude mapping, and **j** polarization angle mapping along [100]_c_.

To further explore the local structure, atomic-resolution scanning transmission electron microscopy (STEM) is used to analyze the local structure of the PNRs. The polarization vectors from the center B-site cations to the corner A-site cations are exhibited by the yellow arrows. As shown in Fig. 2a, the T phase can be clearly confirmed by the arrows with the [001]_c_ direction, according to the 2D Gaussian peak fitting results. The C phase can also be observed by the arrows with almost no polarization magnitude. However, the arrows with the [011]_c_ direction could not distinguish the R and O phases because of their similar projections on the (100)_c_ plane. The detailed transformations of polarization vectors from the T to R/O to C phases are enlarged in Fig. 2b, showing the clear process of a gradual change of polarization. In addition, the magnified image (Fig. 2c) and schematic projection (Fig. 2d) of the unit cell along [100]_c_ show the detailed perovskite structure on the atom scale and the relationship between the direction of the arrows and phases (T and R/O). To further distinguish the R and O phases, atomic-resolution high-angle annular dark-field (HAADF) STEM polarization vector image is performed along [110]_c_. As shown in Fig. 2e, the arrows with of the [1-11]_c_ and [1-10]_c_ directions represent the R and O phases, respectively, and R-O-T-C multiple phases can be obviously observed. The regions with the same polarization direction form PNRs with sizes of ~1–3 nm, which confirms the formation of R-O-T multiphase PNRs coexisting in the C matrix. The R-O-T-C multiphase nanoclusters coexistence strongly destroys the long-range ferroelectric order, resulting in a smaller size of PNRs. It is widely accepted that high activity and external electric field response speed can be provided by small PNRs, which are responsible for low loss and high thermal breakdown strength. Furthermore, the polarization vectors also gradually transform from the T to R to O to C phase (Fig. 2f), which reduces the polarization anisotropy and also leads to an easier polarization response to an external electric field, benefiting the energy efficiency^33^. The magnified image (Fig. 2g) and schematic projection (Fig. 2h) of the unit cell show the perovskite structure in detail and the relationship between the direction of arrows and phases (T, R and O) along [110]_c_. As shown in Fig. 2i, j and Supplementary Fig. 4, the polarization magnitude and polarization angle mappings show an obvious inhomogeneous random distribution state along [100]_c_ and [110]_c_, demonstrating the existence of a strongly perturbed random field, which should be related to the enhanced random stress and electric field caused by the introduction of numerous ions with different ionic radii and valence states, respectively.

### Random oxygen octahedral tilt

**Fig. 3:**
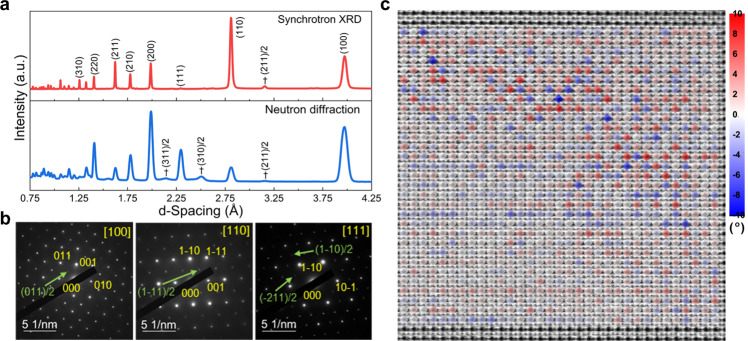
Disordered oxygen octahedron tilt in KNN-H ceramic. **a** Synchrotron XRD and neutron diffraction. “†” represents the positions of the superlattice peaks. **b** SAED patterns along [100]_c_, [110]_c_, and [111]_c_. **c** Atomic-resolution ABF STEM image along [100]_c_ as well as the calculated oxygen octahedral tilt along y axis, blue and red indicate clockwise and anticlockwise tilt, respectively.

In addition to the cation displacement, oxygen octahedral tilt is another important (anti)ferrodistortion. Oxygen octahedral tilt is usually avoided in piezoceramics because it hinders the formation of a textured domain state under an electric field, leading to a large coercive field and poor piezoelectric effects. However, this would be a beneficial factor for designing energy storage capacitors, which would result in delayed polarization saturation. By considering the KNN binary phase diagram drawn by previous researchers, the R, O and T phases in the K_0.2_Na_0.8_NbO_3_ ceramic contain oxygen octahedral tilt (R phase with *a*^−^*a*^−^*a*^−^, O phase with *a*^−^*b*^+^*c*^0^, T phase with *a*^0^*b*^+^*c*^+^ and *a*^0^*a*^0^*c*^+^, and C phase with *a*^0^*a*^0^*c*^+^ and *a*^0^*a*^0^*a*^0^; the +, − and 0 superscripts are Glazer notations for in-phase, anti-phase and no tilt, respectively^34^). Through high-entropy design, there may be multiple types of oxygen octahedral tilt in KNN-H ceramics, which can be directly reflected in the superlattice diffractions. According to the conclusions by Glazer et al., in-phase and anti-phase oxygen octahedral tilt can be identified by {*ooe*}/2 and {*ooo*}/2 (*o* is odd and *e* is even) types of superlattice reflections, respectively^34^. Both {*ooe*}/2 and {*ooo*}/2 superlattice diffractions can be detected in the KNN-H ceramic, as reflected in the synchrotron X-ray diffraction (XRD) and neutron diffraction patterns in Fig. 3a and selected area electron diffraction (SAED) patterns along [100]_c_, [110]_c_, and [111]_c_ in Fig. 3b. However, the local symmetry of PNRs cannot be distinguished by both synchrotron XRD and neutron diffraction patterns owing to the resolution limitation, instead, a pseudocubic phase is usually identified for relaxor ferroelectrics. Therefore, a chaotic local structure with both disordered oxygen octahedral tilt and polarization can be constructed in the KNN-H ceramic, accompanying the movement of the R, T and C phases to the room temperature O phase and the formation of R-O-T-C multiphase nanoclusters, which can be named “local polymorphic distortion”. This conclusion can be directly confirmed by the atomic-resolution annular bright-field (ABF) STEM image along [100]_c_ in Fig. 3c. The deviation of O–O bonds from the *y* axis is used to estimate the oxygen octahedral tilt. Irregular alternations of clockwise and anticlockwise rotation as well as randomly distributed tilt angles can be seen. From the local structure point of view, the studied KNN-H sample can be described as a “hopeless mess”^35^. In addition, as shown in Supplementary Fig. 5, the polarization magnitude and angle mappings for the ABF STEM image of the KNN-H ceramic along [100]_c_ again prove the inhomogeneous state of the polarization distribution.

### Energy storage performance of KNN-H relaxor ceramics

**Fig. 4:**
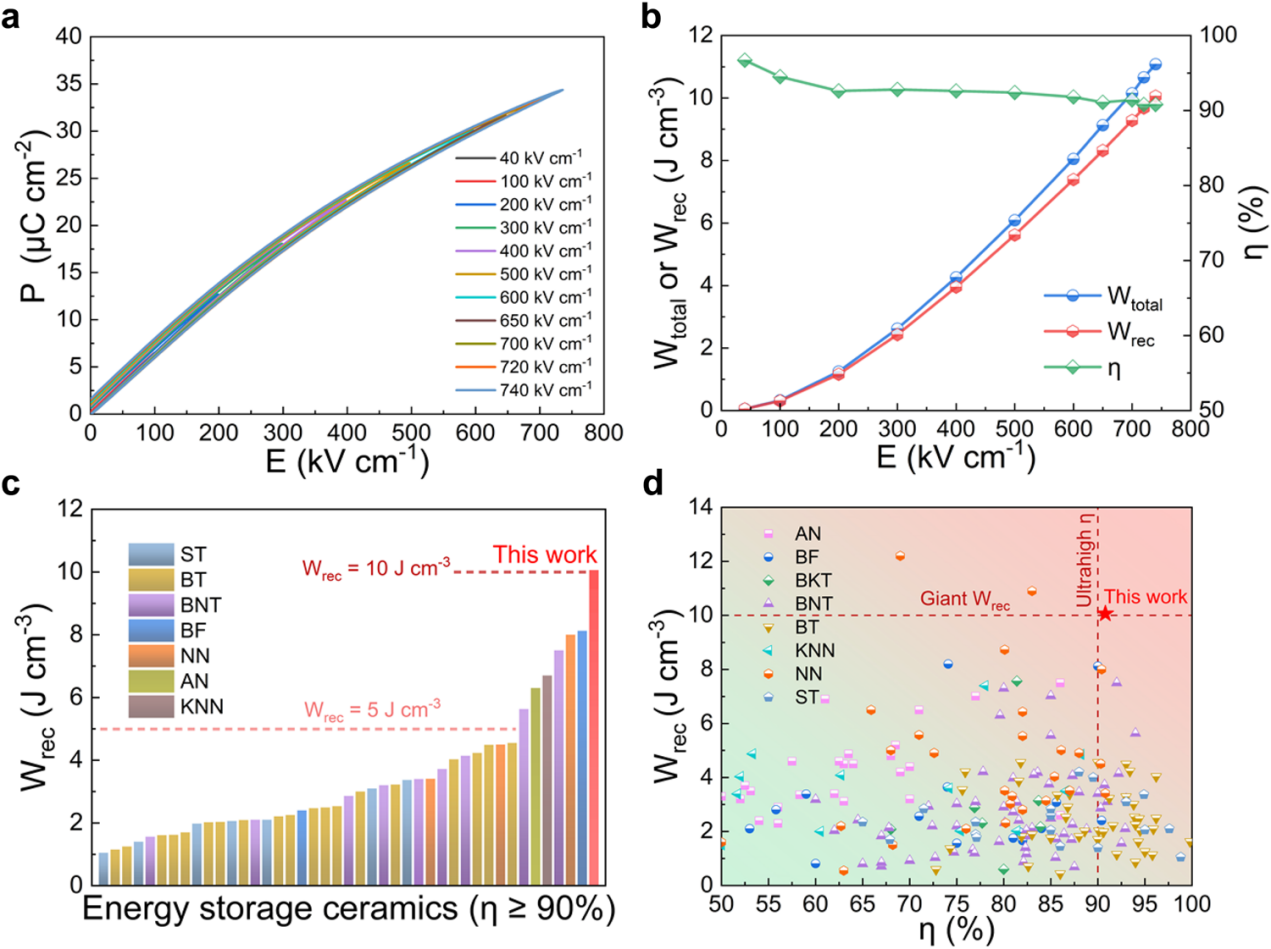
Excellent energy storage performance of KNN-H ceramic. **a** *P*-*E* loops measured till the maximum applied electric fields of KNN-H ceramic. **b** *W*_total_, *W*_rec_, and *η* as a function of *E* for KNN-H ceramic. **c** Comparisons of *W*_rec_ (*η* ≥ 90%) between KNN-H ceramic and other reported lead-free bulk ceramics with *W*_rec_ ≥ 1 J cm^−3^ (ST: SrTiO_3_-based, BT: BaTiO_3_-based). **d** Comparisons of *W*_rec_ versus *η* between KNN-H ceramic and other reported lead-free ceramics (BKT: Bi_0.5_K_0.5_TiO_3_-based).

Ultrahigh comprehensive energy storage performance is necessary for dielectric materials to achieve cutting-edge applications. As shown in Supplementary Fig. 6a, b, the pure KNN ceramics show obvious ferroelectric behavior with poor *W*_rec_ ~0.55 J cm^−3^ and *η* ~54.2%. After introducing the high-entropy strategy to design local polymorphic distortion, the slim *P*-*E* loops of the KNN-H ceramic show particularly low *P*_r_ and polarization hysteresis even under an ultrahigh electric field up to 740 kV cm^−1^. Both the total energy storage density (*W*_total_) and *W*_rec_ show a nearly parabolic growth trend as the applied electric field increases from 40 to 740 kV cm^−1^ (Fig. 4a, b). As a result, a giant *W*_rec_ ~10.06 J cm^−3^ and an ultrahigh *η* ~90.8% are simultaneously achieved in the KNN-H ceramic, showing a significant promotional effect of the high-entropy strategy on the energy storage performance (236% for *E*_b_, 1729% for *W*_rec_, 68% for *η*, Supplementary Fig. 6c). The enhanced *W*_rec_ and *η* benefit from the ultrahigh *E*_b_, large Δ*P* and delayed polarization saturation. Most importantly, Fig. 4c shows that only a few ceramics with energy storage efficiency greater than 90% have broken through the 5 J cm^−3^ level, and the *W*_rec_ of the KNN-H ceramic is approximately more than twice that of most lead-free ceramics, indicating great superiority for low energy consumption. According to Fig. 4d and Supplementary Fig. 7, achieving a giant *W*_rec_ beyond 10 J cm^−3^, especially accompanied by high *η*, is challenging for ceramics. The relevant references can be found in Supplementary Table 1. Significantly, the ultrahigh comprehensive performance (*W*_rec_ ~10.06 J cm^−3^ with *η* ~90.8%) is realized in lead-free bulk ceramics, showing that the bottleneck of ultrahigh energy storage density (*W*_rec_ ≥ 10 J cm^−3^) with ultrahigh efficiency (*η* ≥ 90%) simultaneously in lead-free bulk ceramics has been broken through.

### Hardness, stability and charge/discharge performance to meet applications

**Fig. 5:**
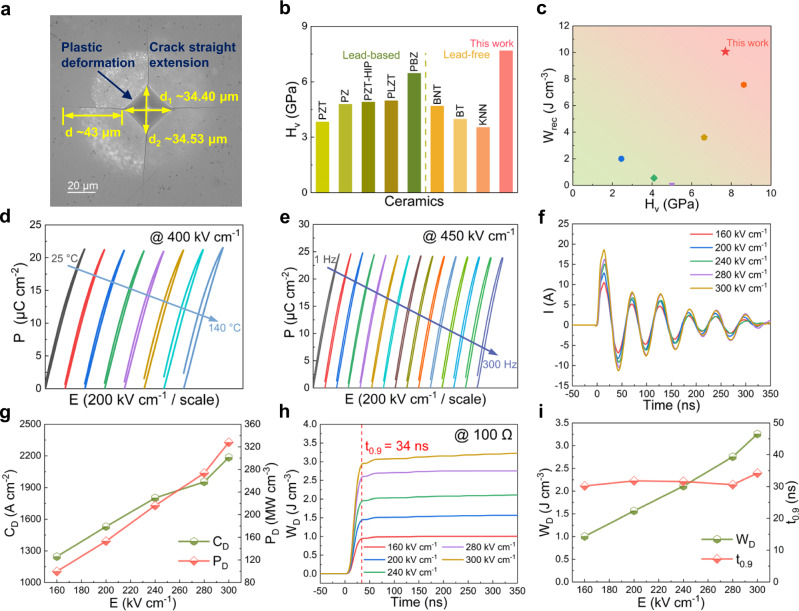
The hardness, stability and charge/discharge performance of KNN-H ceramic. **a** The surface patterns produced by the Vickers diamond indenter. **b** A comparison of *H*_v_ between KNN-H ceramic and some representative lead-free ceramics. **c** A Comparison of *W*_rec_ versus *H*_v_ between KNN-H ceramic and other reported lead-free ceramics. **d** Temperature-dependent *P*-*E* loops at 400 kV cm^−1^. **e** Frequency-dependent *P*-*E* loops at 450 kV cm^−1^. **f** Underdamped discharge waveforms, and **g** *C*_D_, and *P*_D_ values under different electric fields. **h** *W*_D_ as a function of time, and **i** *W*_D_, and *t*_0.9_ values under different electric fields (*R* = 100 Ω).

Mechanical properties such as hardness play an important role in practical applications and directly affect the service life and scope of use of energy storage materials^36,37^. Figure 5a and Supplementary Fig. 8 show the typical patterns produced by a Vickers diamond indenter with a symmetric rhombic indentation for KNN and KNN-H ceramics. The non-plastic deformation transforms into plastic deformation when the high-entropy design is introduced to KNN-H ceramics. An ultrahigh Vickers hardness (*H*_v_) of ~7.70 GPa is obtained for the KNN-H ceramic, which is higher than that of KNN (~3.24 GPa) and some representative perovskite ceramics (Fig. 5b and Supplementary Tables 1–3). Furthermore, the KNN-H ceramic exhibits excellent comprehensive performance in terms of energy storage and hardness (Fig. 5c and Supplementary Table 1), which is helpful for achieving actual applications.

Excellent temperature/frequency/cycling stability of the energy storage performance would give the capacitors an enormous application range^37^. As displayed in Fig. 5d, e, all the *P*-*E* loops of the KNN-H sample are slim with nearly unchanged *P*_max_ under various temperatures from 25 to 140 °C and frequencies from 1 to 300 Hz at 400 and 450 kV cm^−1^, respectively. As a result, temperature (*W*_rec_ ~3.38 ± 0.20 J cm^−3^, *η* ~85.8 ± 6.0%) and frequency (*W*_rec_ ~4.46 ± 0.25 J cm^−3^, *η* ~87.0 ± 4.3%)-insensitive energy storage properties can be achieved, as shown in Supplementary Fig. 9. In contrast to other reported high-performance lead-free capacitors, the KNN-H ceramic exhibits not only high *W*_rec_ but also a broader usage temperature/frequency range. Moreover, when the electric field is cycled up to 10^6^ times, *W*_rec_ and *η* remain almost unchanged under 400 kV cm^−1^, implying the superior cycling stability (Supplementary Fig. 10). All of these make KNN-H ceramics a good candidate for cutting-edge capacitors.

The charge/discharge performance is another key parameter for measuring the potential for applications^11^. Figure 5f, g shows the underdamped discharge property of the KNN-H ceramic under various electric fields. The stable discharge performance can be proven by the regular underdamped oscillating waveforms, which show ultrahigh current density (*C*_D_) ~2186.1 A cm^−2^ and power density (*P*_D_) ~327.9 MW cm^−1^ at 300 kV cm^−1^. The overdamped discharge measurements show an ultrahigh discharge energy density (*W*_D_) ~3.26 J cm^−3^ and an ultrafast discharge rate (*t*_0.9_) ~34 ns at 300 kV cm^−1^ (Fig. 5h, i). According to Supplementary Table 4, the charge/discharge performance also shows obvious superiority compared to other reported ceramics. Furthermore, as shown in Supplementary Fig. 11, the high charge/discharge performance also exhibits good temperature stability from 20 to 160 °C at 260 kV cm^−1^, making KNN-H ceramics ideal for advanced high/pulsed power electronic devices.

## Discussion

The KNN-H ceramic exhibits excellent comprehensive energy storage properties with giant *W*_rec_, ultrahigh *η*, large *H*_v_, good temperature/frequency/cycling stability, and superior charge/discharge performance, showing good prospects for advanced high/pulsed power applications. The achievement of these excellent properties should be ascribed to the ultrahigh *E*_b_, large Δ*P*, delayed polarization saturation and temperature/frequency-stable dielectric response, all of which should originate from the local polymorphic distortion designed by the high-entropy strategy.

First, the KNN-H ceramic shows an ultrahigh *E*_b_. Pure KNN ceramics show quite poor sintering properties and thus usually have quite low relative density^38–40^. An activated lattice as well as a decreased sintering temperature would be realized after high-entropy design by introducing multiple elements, especially because of the important contributions of Li and Bi in KNN-based ceramics to optimization of the sintering properties. As a result, refined grains with a uniform distribution of elements, increased relative density and decreased dielectric loss can be found, as shown in Supplementary Figs. 1–3 and 12. Depletion space charge layers can be built up at the grain boundaries in ceramics, which can act as barriers for the charge carriers transporting across the grain boundaries, leading to high resistivity of the grain boundaries^41^. According to the exponential decay relationship of ${E}_{b}\propto 1/\sqrt{{G}_{a}}$^42^, the increased content of high-resistance grain boundaries would be helpful for the enhanced *E*_b_. In addition, high-bandgap species (Hf and Ta) introduced by high-entropy strategy hinder the transition of electrons from the top of the valence band to the bottom of the conduction band, thereby enhancing the intrinsic *E*_b_^10,37,43^. The impedance performance of the studied samples is measured from 300 to 450 °C and in the frequency range of 50 Hz to 2 MHz to analyze impedance contributions. As shown in Supplementary Fig. 13, the resistance value at 400 °C of KNN-H is larger than that of KNN, which is mainly related to the increase in the grain boundaries and is responsible for the enhanced *E*_b_. Furthermore, the formation of coexisting R-O-T-C multiphase nanoclusters can effectively reduce the size of PNRs and loss, greatly decreasing the possibility of thermal breakdown. Therefore, the reduction of grain size to submicron (*G*_a_ ~600 nm) with a uniform and dense structure and R-O-T-C multiphase nanoclusters originating from the high-entropy design should mainly contribute to the large enhancement of *E*_b_ from 220 to 740 kV cm^−1^.

Second, the KNN-H ceramic has a large Δ*P* ~32.7 μC/cm^2^, which should be related to both large *P*_max_ and near-zero *P*_r_. On the one hand, PNRs with different symmetries coexisting in the nonpolar matrix via high-entropy strategy can make the flexible polarization reorientation process with small stress under electric field, leading to the enhanced polarization texture along the direction of the electric field and providing the basic for large *P*_max_. Moreover, the introduction of Bi by high-entropy strategy would also enhance polarization due to the orbital hybridization between Bi 6*s* and O 2*p*^36,37^. On the other hand, the large random field in the ergodic relaxor region with coexisting R-O-T-C multiphase nanoclusters in this high-entropy ceramic would drive the long-range ordering ferroelectric state back to the initial macro nonpolar state during unloading, resulting in a near-zero *P*_r_.

Third, the delayed polarization saturation also plays an important role in the excellent energy storage properties of the studied KNN-H ceramic. Weak correlation between PNRs owing to the large random electric field, which mainly correlates with the large compositional disorder of the nature of high-entropy materials, would delay a polarization texture along the electric field direction. The medium *ε*_r_ ~550 at room temperature controlled by high-entropy strategy can also effectively delay polarization saturation. At the same time, a unique structure of randomly distributed oxygen octahedral tilt can be found in this KNN-H sample. When an external electric field is applied, the tilt distortion of the oxygen octahedron causes some electric energy to be absorbed during the process of forming long-range ferroelectric ordering, resulting in delayed polarization saturation. In a word, the complex inhomogeneous local distortion structure leads to nearly linear *P-E* loops with a small slope for the studied high-entropy ceramic.

Fourth, the ultrahigh hardness should be contributed by the ultrafine grains with a dense microstructure^44^, mass disorder and solid solution hardening caused by high-entropy design^26^, which can withstand the compressive forces generated by the electrostatic attraction of the surface charges and stress generated by the electrostriction effect to reduce the possibility of electromechanical breakdown.

Lastly, the enhanced dielectric relaxation behavior through entropy enhancement should benefit both the temperature and frequency stability. The enhanced entropy would broaden the distribution of the *T*_c_ value of each PNR. In addition, the oxygen octahedral tilt system shows good temperature stability, as confirmed by the stable superlattice and unchanged local structure information in Supplementary Figs. 14 and 15. As a result, a temperature-stable dielectric response meeting the standard of X8R capacitors can be achieved for the KNN-H ceramic, as shown in Supplementary Fig. 1. Taking into consideration the near-linear *P*-*E* response of the studied sample, temperature-stable energy storage can be ensured. Moreover, the strong entropy weakens the correlation between PNRs, leading to a fast response of each PNR under an electric field and bringing about excellent frequency stability.

In summary, a high-entropy strategy is proposed to design “local multiple distortion” including R-O-T-C multiphase nanoclusters coexistence and random oxygen octahedral tilt distortion for lead-free relaxors to enhance the comprehensive energy storage performance, leading to ultrasmall PNRs, ultrafine grains with a dense microstructure, and delayed polarization saturation. A giant *W*_rec_ ~10.06 J cm^−3^ with an ultrahigh *η* ~90.8% is realized in lead-free relaxor ferroelectrics, which is the optimal comprehensive energy storage performance reported to date for lead-free bulk ceramics, showing a breakthrough progress. The excellent mechanical properties, charge/discharge performance and stability of KNN-H ceramics also show great potential for use in energy storage capacitors. It is encouraging that a new avenue is opened up for designing ultrahigh comprehensive energy storage performance, meeting the urgent demand for advanced high-power or pulsed power capacitors.

## Methods

### Sample preparation

(K_0.2_Na_0.8_)NbO_3_ (KNN) and [(K_0.2_Na_0.8_)_0.8_Li_0.08_Ba_0.02_Bi_0.1_](Nb_0.68_Sc_0.02_Hf_0.08_Zr_0.1_Ta_0.08_Sb_0.04_)O_3_ (KNN-H) ceramics were fabricated by a conventional solid-state reaction method. K_2_CO_3_ (Aladdin, 99.99%), Na_2_CO_3_ (Aladdin, 99.99%), Nb_2_O_5_ (Aladdin, 99.9%), Li_2_CO_3_ (Aladdin, 99.99%), BaCO_3_ (Aladdin, 99.95%), Bi_2_O_3_ (99.99%), Sc_2_O_3_ (Macklin, 99.0%), HfO_2_ (Macklin, 99.99%), ZrO_2_ (Macklin, 99.99%), Ta_2_O_5_ (Aladdin, 99.9%), and Sb_2_O_3_ (Macklin, 99.5%) were used as the starting materials. The stoichiometric powders were planetary ball-milled with alcohol in nylon jars for 24 h using yttrium stabilized zirconia balls as milling media. The mixed powers were dried at 120 °C for 2 h and then calcined at 800 °C for 5 h. Then, the 0.5 wt% PVB binder was mixed with the as-synthesized powders by high-energy ball milling (600 r/min for 15 h) with alcohol. After drying at 120 °C for 2 h, the mixed powders were pressed into pellets with diameters of 1 cm under 300 Mpa. The pellets were heated to 550 °C at 3 °C/min to burn out the binder and then sintered at 1230 °C in closed crucibles for 2 h. The ceramics were polished into a thickness of ~0.006–0.010 cm for electrical property tests. Two parallel surfaces were coated with silver electrodes with an area of ∼0.00785 cm^2^ (∼0.1 cm in diameter), which were fired at 550 °C for 30 min.

### Structure characterizations

The crystal structure was characterized by powder neutron diffraction using time-of-flight powder diffractometers collected at CSNS (China Spallation Neutron Source, MPI) and high-energy synchrotron XRD (*λ* = 0.1173 Å) with a beam size of 0.05 cm × 0.05 cm conducted at the 11-ID-C beamline of the Advanced Photon Source. Temperature-dependent XRD tests were performed using an X-ray diffractometer (X’pert PRO, PANalytical, the Netherlands). Temperature-dependent Raman spectra were tested on well-polished samples under 532 nm excitation using a Raman scattering spectrometer (Horiba Jobin Yvon HR800, France) with a heating stage (Linkam, THM 600, UK). The morphology of grains and element distribution maps of samples were analyzed using a SEM (LEO1530, ZEISS SUPRA 55, Oberkochen, Germany). The samples were carefully polished to 0.004 cm and then prepared by an ion milling system (PIPS, Model 691, Gatan Inc., Pleasanton, CA, USA) with a liquid nitrogen cooled stage for TEM measurement. Domain morphology and SAED were observed on a field-emission TEM (JEM-F200, JEOL, Japan) at an accelerating voltage of 200 kV. HAADF and ABF atomic-scale images were acquired using an atomic-resolution STEM (aberration-corrected Titan Themis G2 microscope). Accurate atomic positions in the STEM images were clarified by 2D Gaussian fitting. The polarization vector, polarization magnitude and polarization angle maps were calculated by customized MATLAB scripts.

### Electrical property measurements

The room temperature *P*-*E* loops with test frequency of 10 Hz and temperature-, frequency- and cycle-dependent *P*-*E* loops were measured using a ferroelectric analyzer (aix ACCT, TF Analyzer 1000, Aachen, Germany). Temperature- and frequency-dependent dielectric performance and impedance spectra were performed using a precision LCR meter (Keysight E4990A, Santa Clara, CA). The charge/discharge properties of samples with a thickness of ~0.008 cm were conducted using a commercial charge–discharge platform (CFD-001, Gogo Instruments Technology, Shanghai, China).

### Mechanical performance measurements

The Vickers hardness of the well-polished samples with a thickness of ~0.1 cm were performed under a load of 4.9033 N for 15 s using a Vickers diamond indenter (FALCON 507, INNOVATEST, the Netherlands) and recorded through a metallurgical microscope (DMi 8C, Leica, Germany).

## Supplementary information

Supplementary Information

## Data Availability

All data supporting this study and its findings are available within the article and its Supplementary Information. Any data deemed relevant are available from the corresponding author upon request.
