# Supplementary material for: Giant energy-storage density with ultrahigh efficiency in lead-free relaxors via high-entropy design
Source: Nat Commun. 2022 Jun 2;13:3089. doi: 10.1038/s41467-022-30821-7 (PMC9163056; doi:10.1038/s41467-022-30821-7)
Supplement: Supplementary file 1 — Supplementary Information [file 41467_2022_30821_MOESM1_ESM.pdf]

Supplementary Information

Giant energy-storage density with ultrahigh efficiency in lead-free relaxors via high-entropy design

Liang Chen[1,2], Shiqing Deng[1,3], Hui Liu[1,3], Jie Wu[3], He Qi[1,2]*, and Jun Chen[1,2]*

[1]Beijing Advanced Innovation Center for Materials Genome Engineering, University of Science and Technology Beijing, Beijing 100083, China

[2]Department of Physical Chemistry, University of Science and Technology Beijing, Beijing 100083, China

[3]School of Mathematics and Physics, University of Science and Technology Beijing, Beijing 100083, China

*Corresponding author, E-mail: qiheustb@ustb.edu.cn; junchen@ustb.edu.cn

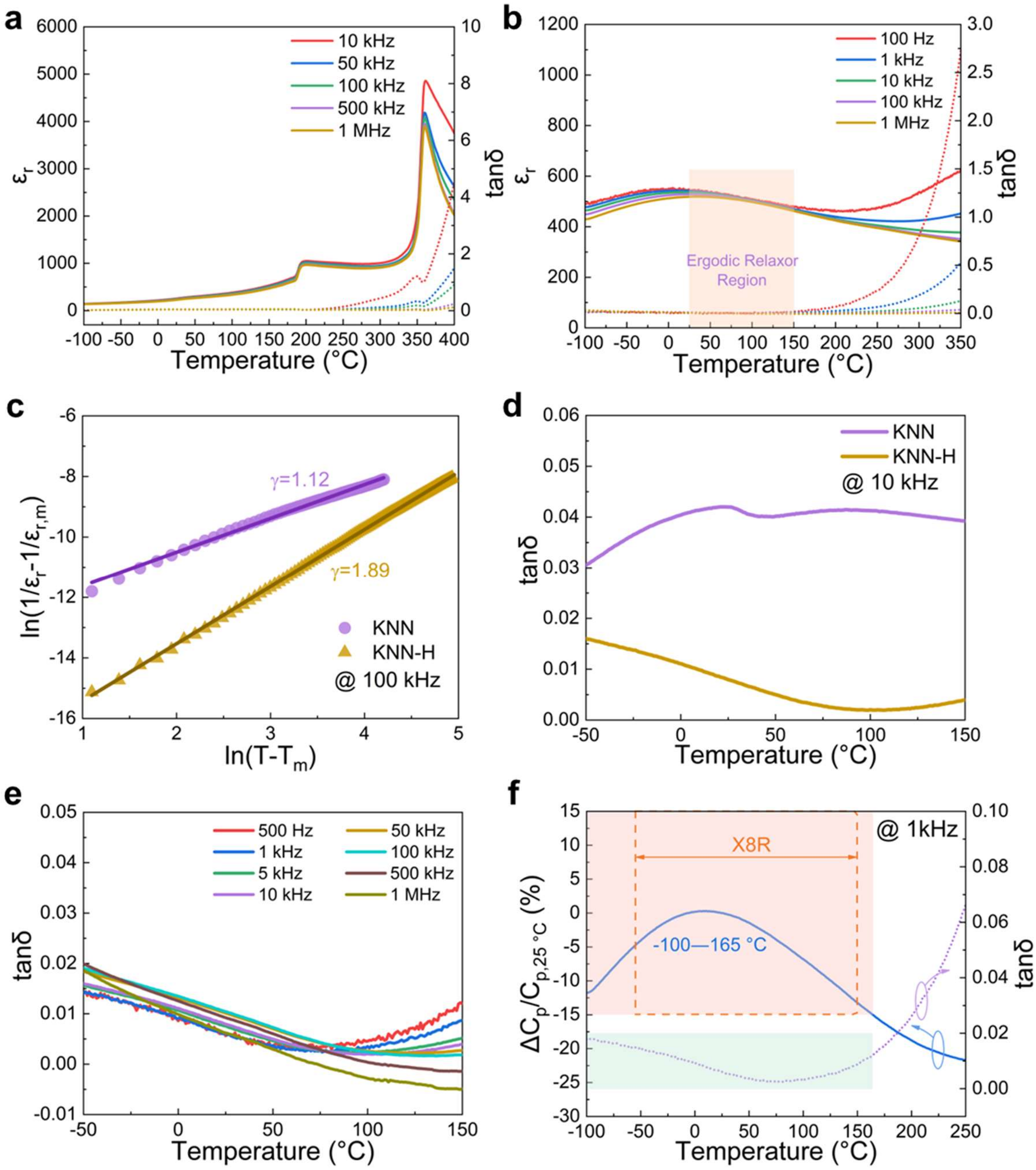

Supplementary Fig. 1 | The dielectric performance of KNN and KNN-H ceramics. Dielectric constant ε_r and dielectric loss tanδ as a function of temperature at various frequencies for **a** KNN and **b** KNN-H ceramics. **c** $\ln(1/\varepsilon_r - 1/\varepsilon_{r,m})$ vs. $\ln(T\text{-}T_m)$ at 100 kHz and **d** tanδ as a function of temperature at 10 kHz for KNN and KNN-H ceramics. **e** tanδ as a function of temperature at various frequencies for KNN-H ceramic. **f** $\Delta C_p/C_{p,25°C}$ and dielectric loss tanδ as a function of temperature at 1 kHz for KNN-H ceramic. It can be seen that KNN ceramic shows normal ferroelectric O phase at room temperature, existing clear phase transition of O-T and T-C at about 195 and 360 °C, respectively.

The dielectric relaxation behaviors with obvious frequency dispersion and diffusion phase transition are found in KNN-H ceramic, which is located in a ergodic relaxor region above room temperature. It is recognized that PNRs rather than domains or nano-domains mainly exist in the ergodic relaxor region, which are helpful for achieving an ultrahigh η in energy storage materials.[1] Furthermore, the medium ε_r ~550 can effectively delay polarization saturation and reduce the probability of electromechanical breakdown due to the weak electrostrictive effect. The diffusion coefficient γ can be calculated by the modified Curie-Weiss law:

$$\frac{1}{\varepsilon_r} - \frac{1}{\varepsilon_{r,m}} = \frac{(T-T_m)^\gamma}{C} \quad (1)$$

where ε_r is the dielectric permittivity, $\varepsilon_{r,m}$ is the maximum dielectric constant, T_m is the corresponding temperature of the $\varepsilon_{r,m}$, and C is the Curie constant.[2] The γ value of a normal ferroelectrics is 1, and $\gamma = 2$ for an ideal relaxor ferroelectric. Compared with KNN ceramic (γ ~1.12), the KNN-H ceramic designed by the high-entropy strategy have higher γ (~1.89), exhibiting enhanced dielectric relaxation characteristics. Importantly, compared with KNN ceramic, the tanδ of KNN-H ceramic is reduced by about 5 times from -50 to 150 °C, which is mainly attributed by the effective high-entropy design. The tanδ of KNN-H ceramic can be effectively maintained below 0.02 in a wide temperature (-50-150 °C) and frequency (0.5-1000 kHz) range, showing the excellent low loss characteristics. These can largely improve E_b owing to the increase of thermal breakdown strength. In addition, the capacitance C_p of KNN-H ceramic can be effectively maintained within ± 15% with low loss (< 0.02) from -100 to 165 °C, meeting the demanding requirements of X8R capacitors (± 15%, -50-150 °C). All of these show that KNN-H ceramic exhibits a great potential in advanced high/pulsed power capacitors.

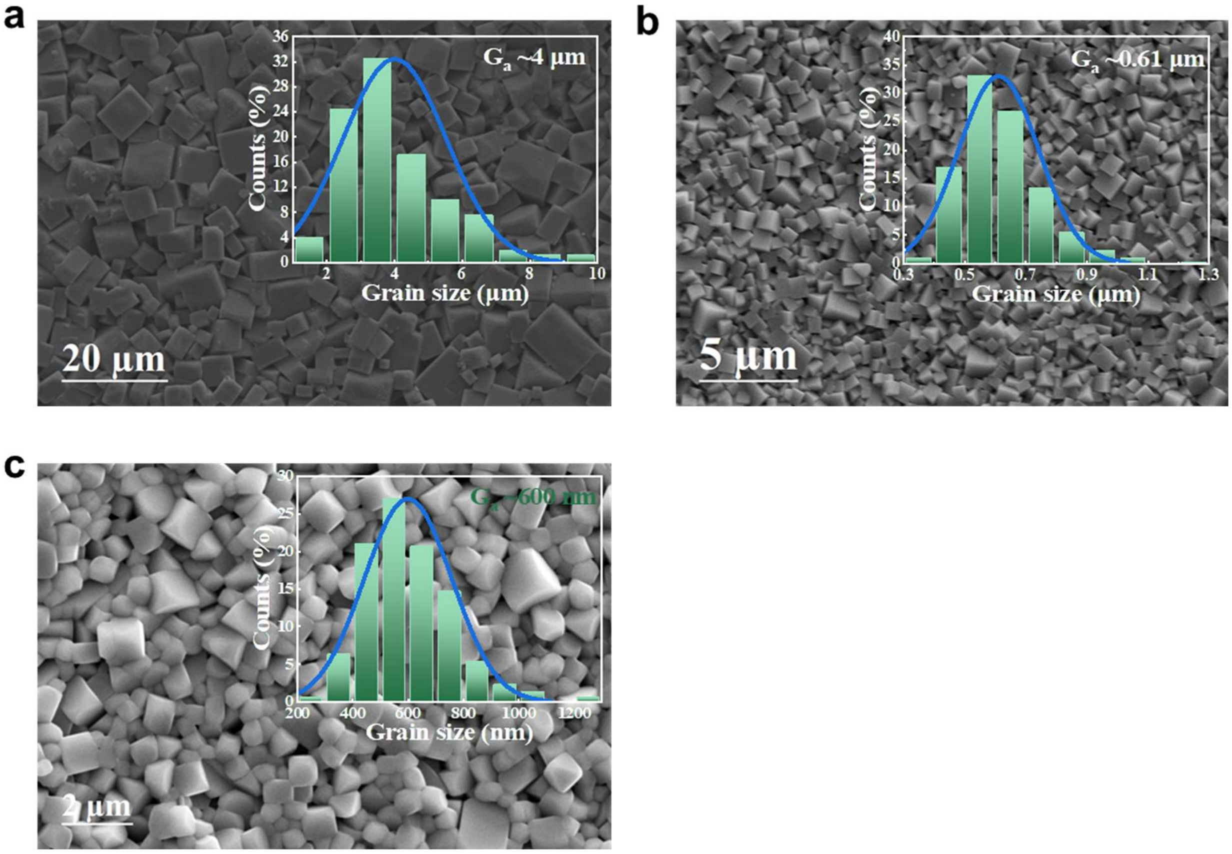

Supplementary Fig. 2 | The SEM morphology and grain size distribution of KNN and KNN-H ceramics. **a** SEM surface morphology and grain size distribution of KNN ceramic. **b** SEM surface morphology and grain size distribution of KNN-H ceramic. **c** SEM morphology and grain size distribution of the thermally etched KNN-H ceramic after polishing. It can be seen that large holes exist in KNN ceramic, which may withstand higher electric field and bring the short plate effect of rapid breakdown. Compared with the microstructure of KNN ceramics, KNN-H ceramics are denser with less porous, and the grain size distribution is more uniform. The average grain size (G_a) also greatly decreases from 4 (KNN) to 0.61 μm (KNN-H) when the high-entropy strategy is adopted to KNN ceramic. Furthermore, the internal grain size (~600 nm) of KNN-H ceramic is almost identical to that of the surface, showing a homogeneous and dense microstructure in the KNN-H sample.

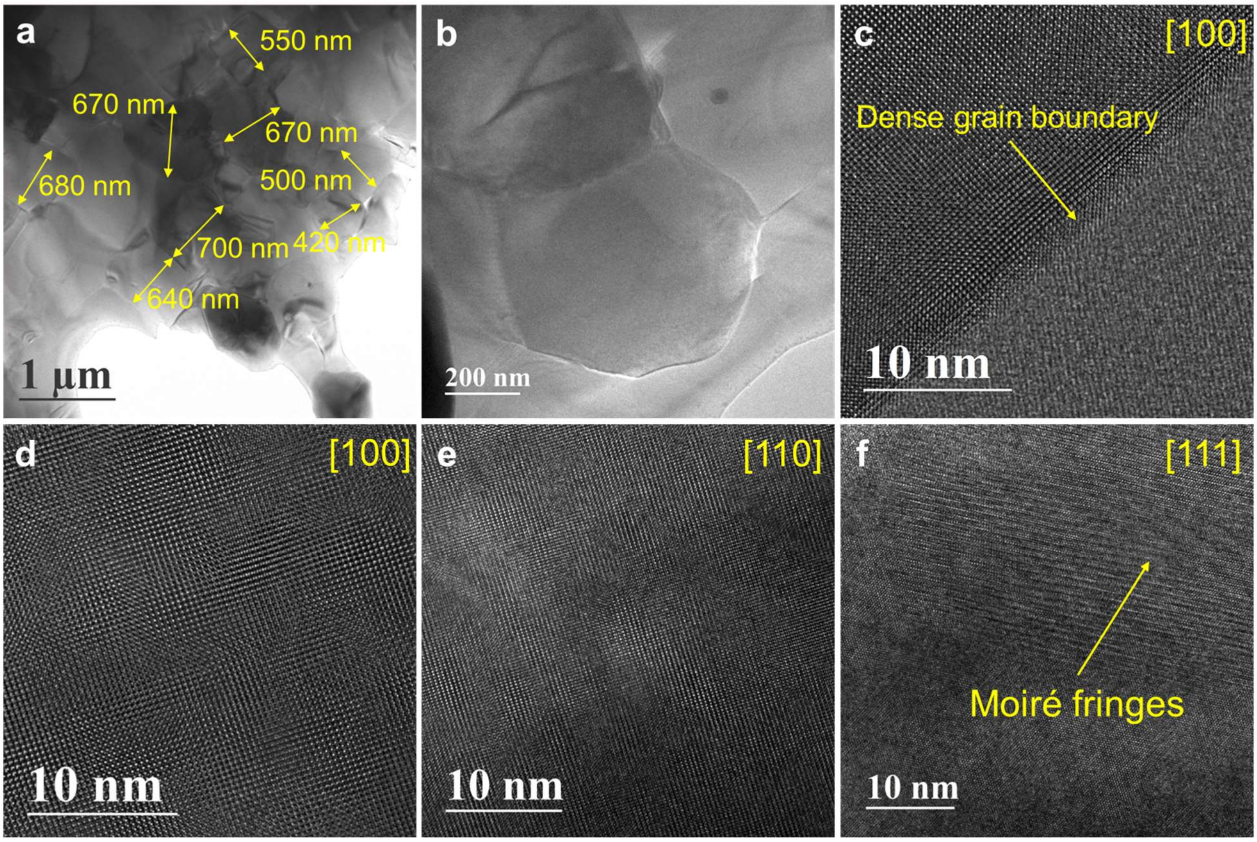

Supplementary Fig. 3 | TEM images of KNN-H ceramic. a Grain. b Grain boundary. c HR-TEM of grain boundary. Domain morphology along d $[100]_c$, e $[110]_c$, and f $[111]_c$. Ultrafine grain size (~400-700 nm) and dense grain boundary without pores can be clearly observed in KNN-H ceramic, showing a dense microstructure and an excellent sample quality, which are highly consistent with the SEM results. However, no obvious nanodomains or PNRs morphology can be observed by HR-TEM along $[100]_c$, $[110]_c$, and $[111]_c$ due to the insufficient resolution. The presence of Moiré fringe structure originated from the interference of two overlaid lattice patterns with mismatched orientations can imply the composition fluctuations on micro scale, supporting the formation of PNRs in a complex perovekite material.[3] However, the existence of the PNRs still needs to be further confirmed.

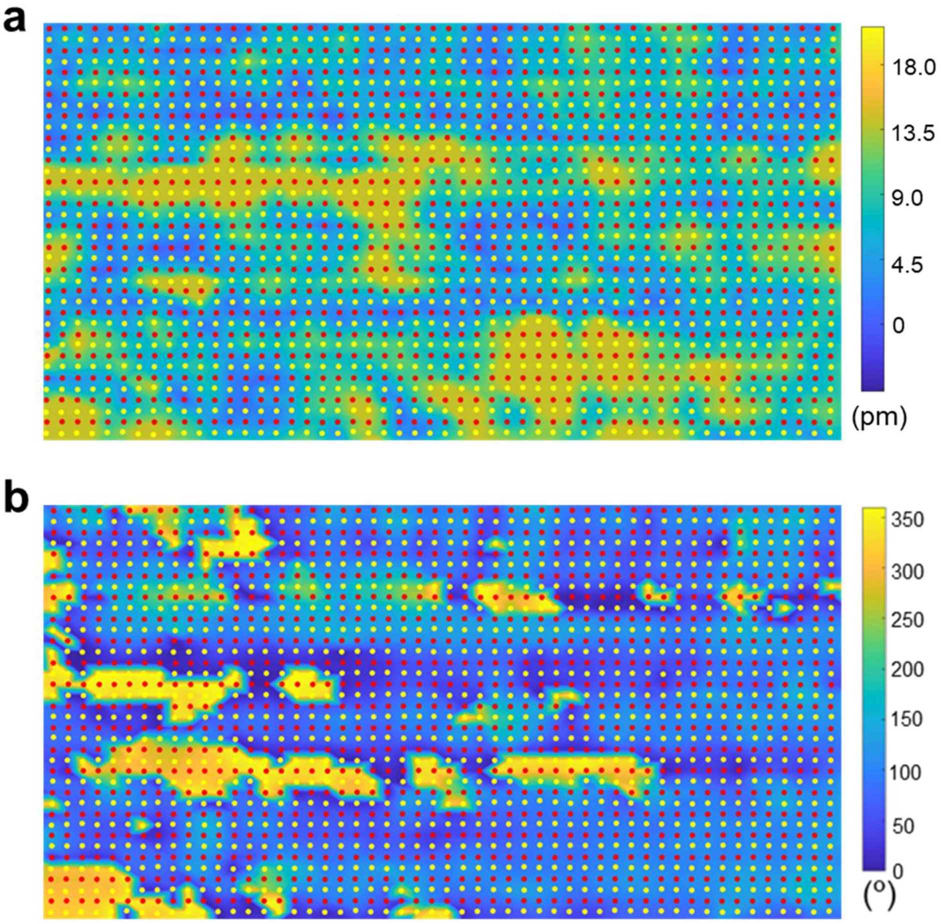

Supplementary Fig. 4 | Polarization magnitude and angle mappings for the HAADF STEM polarization vector image along $[110]_c$. **a** Polarization magnitude mapping. **b** Polarization angle mapping. Both the polarization magnitude and angle mappings show obvious inhomogeneous random distribution state. The inhomogeneous random distribution state mainly originates from the enhanced random stress field and random electric field when numerous ions with different ionic radii and valence states are introduced into the KNN lattices.

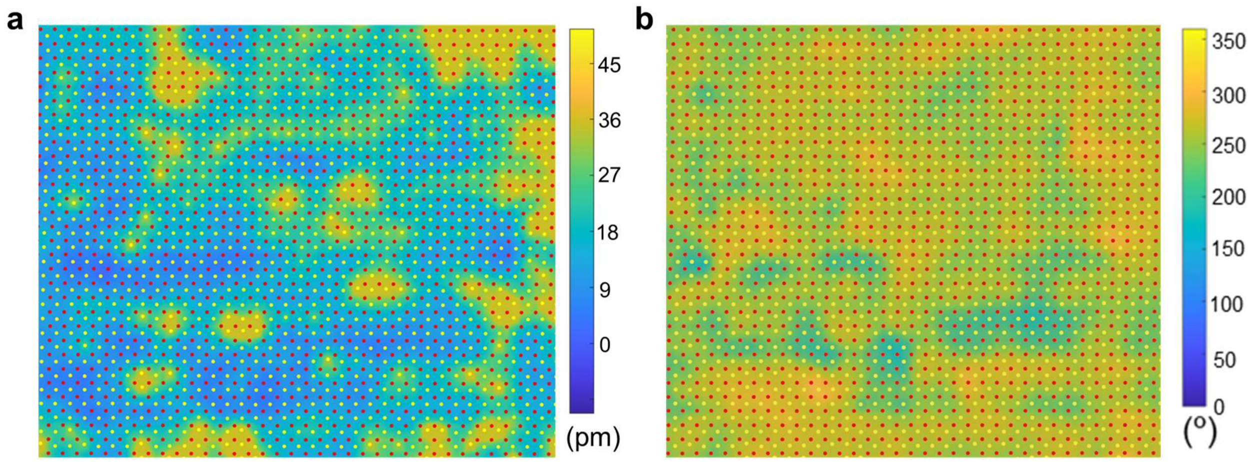

Supplementary Fig. 5 | Polarization magnitude and angle mappings for the ABF STEM of KNN-H ceramic along [100]$_c$. a Polarization magnitude mapping. **b** Polarization angle mapping. It is obvious that both the polarization magnitude and angle mappings show inhomogeneous random distribution state.

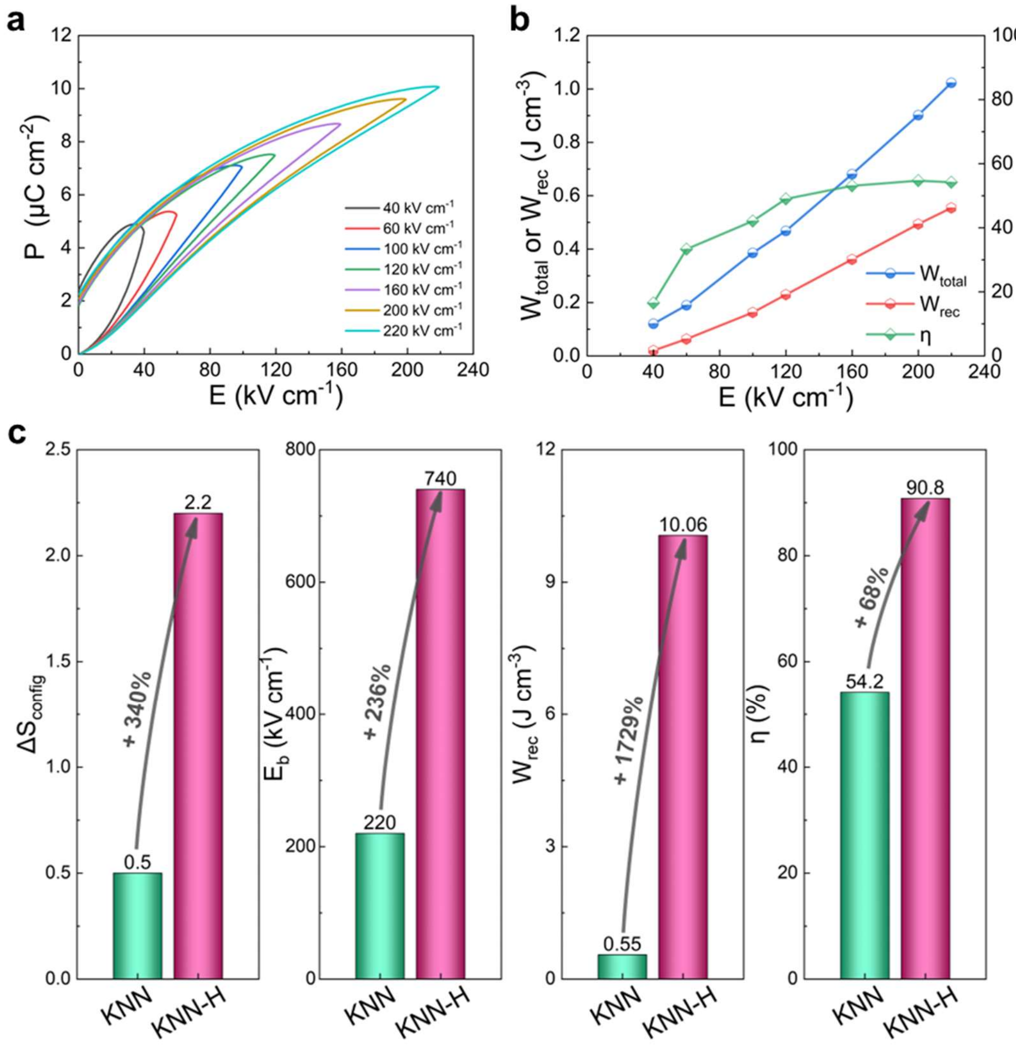

Supplementary Fig. 6 | The energy storage performance of KNN and KNN-H ceramics. **a** *P*-*E* loops measured till the maximum applied electric fields and **b** W_{total}, W_{rec}, and η as a function of E for KNN ceramic. **c** Comparisons of ΔS_{config}, E_b, W_{rec} and η between KNN and KNN-H ceramics. Pure KNN ceramic shows the obvious normal ferroelectric behaviors from the *P*-*E* loops with large hysteresis and P_r under electric fields from 40 to 220 kV cm^{-1}. Both the energy storage density and efficiency gradually increase with increasing electric field. As a result, low energy storage properties (W_{rec} ~0.55 J cm^{-3} and η ~54.2%) are obtained in KNN ceramic at 220 kV cm^{-1}, which are mainly related to the low E_b and large polarization hysteresis. In perovskites, the configurational entropy (ΔS_{config}) can be calculated using the following formula:

$$\Delta S_{config} = -R[(\sum_{a=1}^{n} x_a \, lnx_a)_{A-site} + (\sum_{b=1}^{n} x_b \, lnx_b)_{B-site} + 3(\sum_{c=1}^{n} x_c \, lnx_c)_{O-sit} \ ] \qquad (2)$$

where x_a, x_b and x_c are the mole fraction of the ions present in the A-site, B-site and O-site, respectively.[4] When $\Delta S_{config} < 0.69R$, it is a low-entropy material. It belongs to medium-entropy material when $0.69R \leq \Delta S_{config} < 1.61R$. When $\Delta S_{config} \geq 1.61R$, it is a high-entropy material. According to Eq. (2), the ΔS_{config} of KNN and KNN-H ceramics are 0.5 and 2.2, respectively, obviously showing that the KNN and KNN-H ceramics belong to low-entropy and high-entropy materials, respectively. A significantly increase of energy storage performance (236% for E_b, 1729% for W_{rec}, 68% for η) is observed when ΔS_{config} increases from 0.5 (KNN ceramic) to 2.2 (KNN-H ceramic), proving that high-entropy strategy can effectively improve the comprehensive properties in energy storage materials.

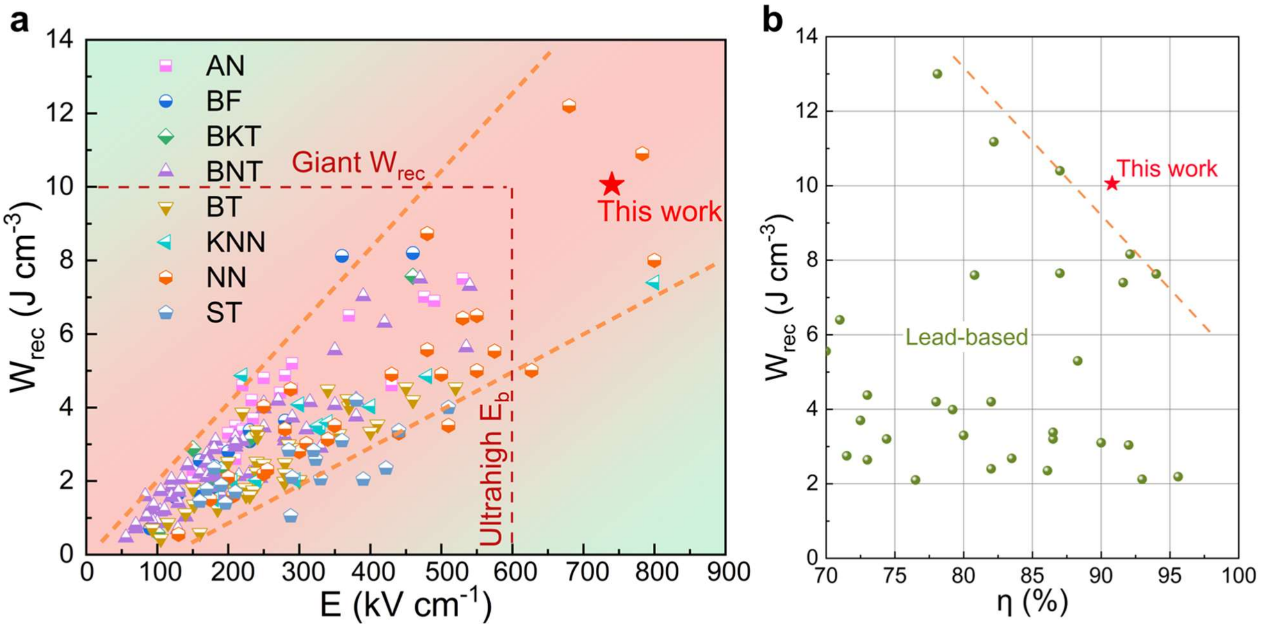

Supplementary Fig. 7 | Comparisons of energy storage performance. a Comparisons of W_{rec} versus E between KNN-H ceramic and other reported lead-free ceramics. b Comparisons of W_{rec} versus η between KNN-H ceramic and other reported lead-based ceramics. The summary of W_{rec} versus E in lead-free ceramics shows that high energy storage density is inseparable from large E_{b}. Compared with other reported lead-free ceramic systems, KNN-H ceramics exhibit great breakdown resistance (ultrahigh E_{b} ~740 kV cm^{-1}) and excellent energy storage performance. In addition to lead-free ceramics, the comprehensive properties of KNN-H ceramics also show great superiority in lead-based ceramics and also have the advantages of environmental protection. In summary, the ultrahigh comprehensive energy storage properties of KNN-H ceramic are realized in lead-free bulk ceramics, demonstrating breakthrough progress in the field of energy storage.

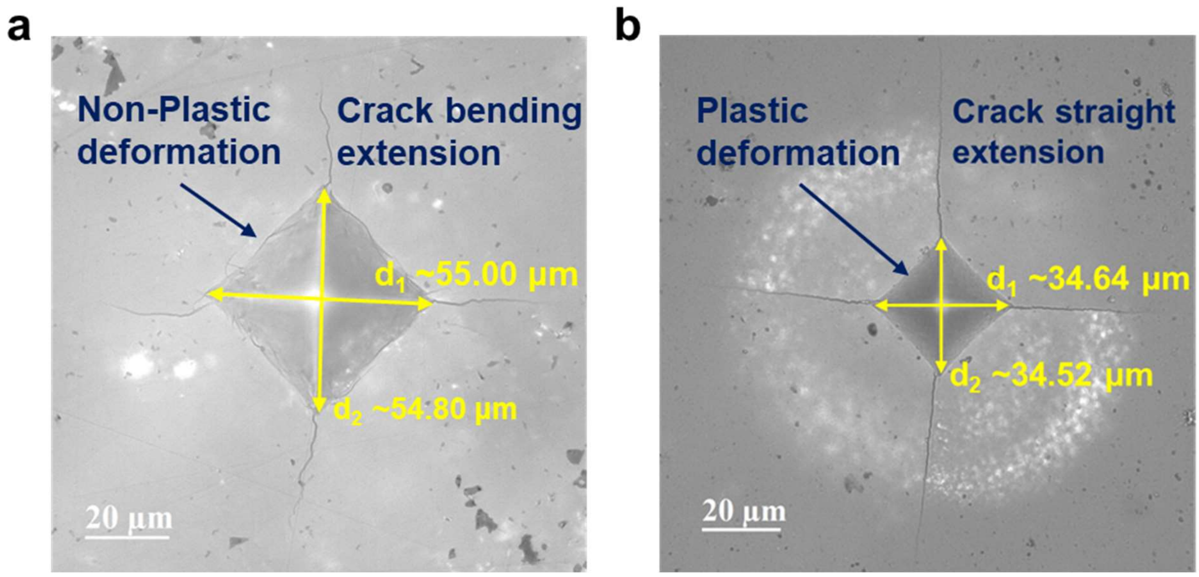

Supplementary Fig. 8 | The hardness performance of KNN and KNN-H ceramics. The surface patterns produced by the Vickers diamond indenter for **a** KNN and **b** KNN-H ceramics. It is obvious that non-plastic deformation with cracks bending extension generated in KNN ceramic. The clear plastic deformation with complete diamond-shaped pits and straight extension can be found in KNN-H ceramic, showing the excellent mechanical performance of hardness. The Vickers hardness H_v can be calculated as:

$$H_v = 1.8544\frac{F}{d^2} \qquad (3)$$

where F is the applied load of 4.9033 N, and d is the average value of d_1 and d_2.[5] As a result, an ultrahigh Vickers hardness (~7.70 Gpa) can be obtained in KNN-H ceramic, which is more than twice that of KNN ceramics (~3.24 Gpa).

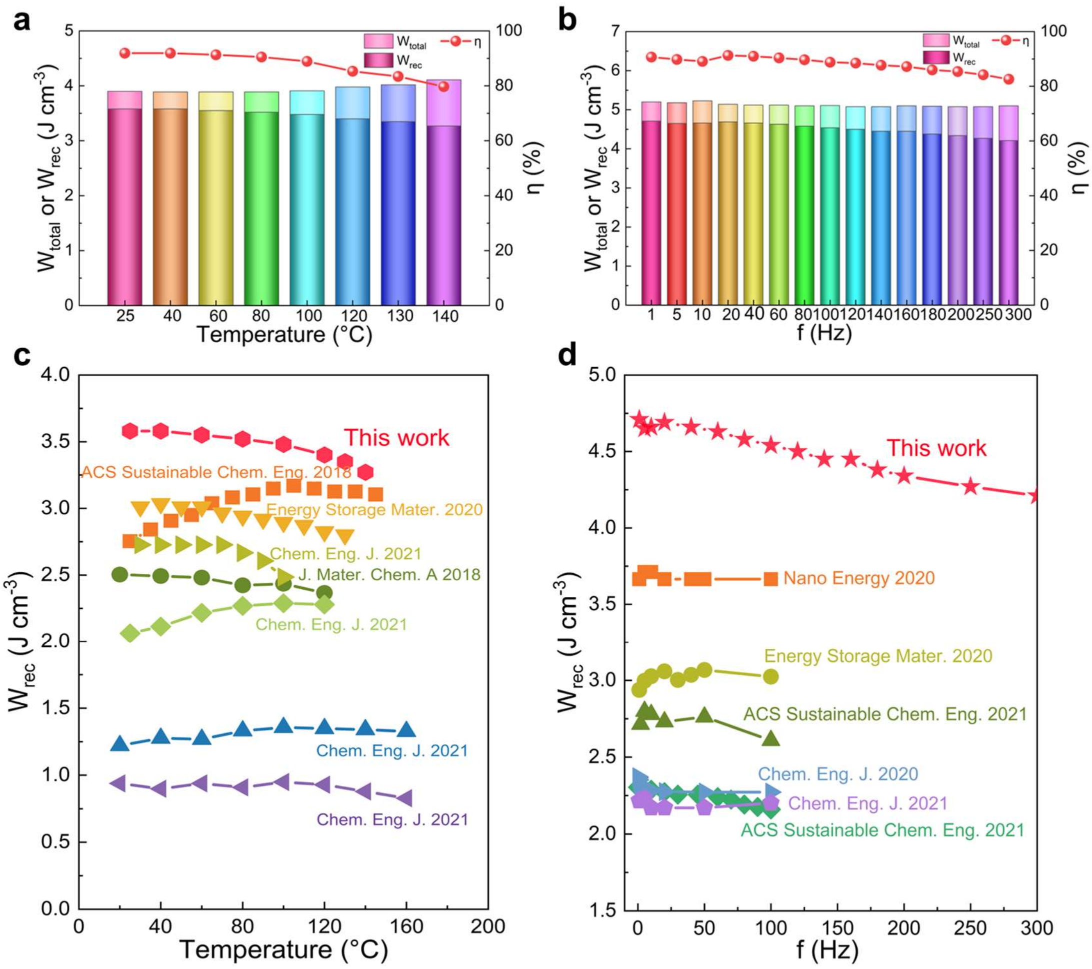

Supplementary Fig. 9 | Temperature and frequency stability of energy storage performance for KNN-H ceramics. **a** W_{total}, W_{rec} and η as a function of temperature under 400 kV cm^{-1}. **b** W_{total}, W_{rec} and η as a function of frequency under 450 kV cm^{-1}. Comparisons of energy storage performance in wide operating **c** temperature and **d** frequency range between KNN-H ceramic and other reported ceramics. When the temperature increase from 25 to 140 °C, the energy storage performance (W_{rec} ~3.38 ± 0.20 J cm^{-3}, η ~85.8 ± 6.0%) of KNN-H ceramic can be well maintained at a high electric field of 400 kV cm^{-1}, showing good temperature stability. For frequency stability, excellent energy performance (W_{rec} ~4.46 ± 0.25 J cm^{-3}, η ~87.0 ± 4.3%) are observed in KNN-H ceramics from 1 to 300 Hz at 450 kV cm^{-1}, resulting in frequency-insensitive energy storage performance. Moreover, the comparisons of temperature and frequency stability are conducted in Supplementary Fig. 9c and d, respectively. It should be noted that the KNN-H ceramic not only exhibits higher W_{rec} but also exhibit a broader usage temperature/frequency range than other representative lead-free ceramics, making it a good candidate for cutting-edge capacitors.

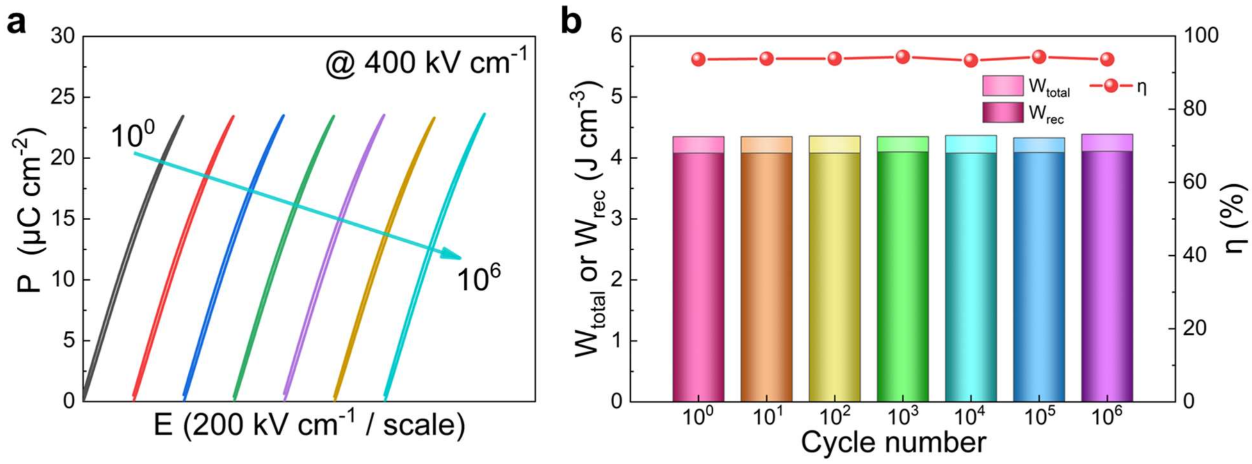

Supplementary Fig. 10 | Cycling stability of energy storage performance for KNN-H ceramics. **a** *P*-*E* loops, and **b** W_{total}, W_{rec} and η as as a function of the cycle number under an electric field of 400 kV cm^{-1}. When the electric field is cycled up to 10^6 times, *P*-*E* loops remain almost unchanged. As a result, cycle-insensitive energy storage properties (W_{rec} ~4.10 ± 0.02 J cm^{-3}, η ~94.0 ± 0.4%) can be achieved.

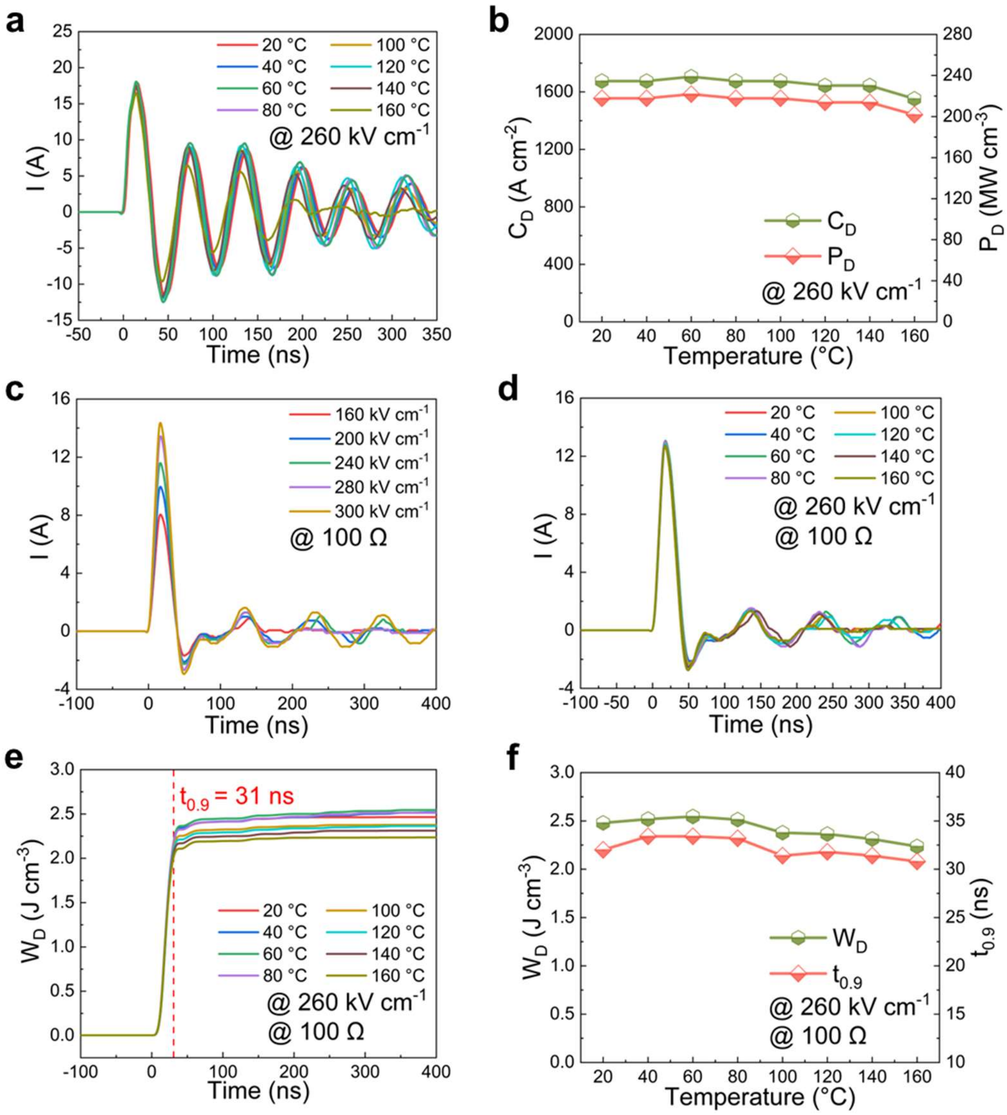

Supplementary Fig. 11 | The charge/discharge performance of KNN-H ceramic. **a** Underdamped discharge waveforms and **b** C_D, and P_D values at 260 kV cm^{-1} under different temperature. Overdamped discharge waveforms under **c** different electric fields and **d** different temperature. **e** W_D as a function of time, and **f** W_D, and $t_{0.9}$ values at 260 kV cm^{-1} under different temperature (R = 100 Ω). The current density C_D, power density P_D and discharge energy density W_D can be calculated using the following formulas:

$$C_D = \frac{I_{max}}{S} \quad (4)$$

$$P_D = \frac{E \times I_{max}}{2S} \quad (5)$$

$$W_D = \frac{R\int I_{(t)}^2 dt}{V} \quad (6)$$

where S and V is the electrode area and sample volume, respectively. The stable discharge performance can be proved by regular underdamped oscillating waveforms at different temperature from 20 to 160 °C. The C_D (≥ 1552.4 A cm^{-2}) and P_D (≥ 201.8 MW cm^{-3}) can be effectively maintained in wide temperature range at 260 kV cm^{-1}. The overdamped discharge performance is performed with a fixed load resistance (R = 100 Ω) at various electric fields from 160 to 300 kV cm^{-1} and temperatures from 20 to 160 °C, showing stable overdamped oscillating waveforms and ultrahigh discharge rate. The W_D (≥ 2.24 J cm^{-3}) and $t_{0.9}$ (≤ 34 ns) can be well kept in wide temperature range from 20 to 160 °C at 260 kV cm^{-1}. As a result, KNN-H ceramic exhibits excellent charge/discharge performance and stability, making good prospects for advanced pulsed-power applications.

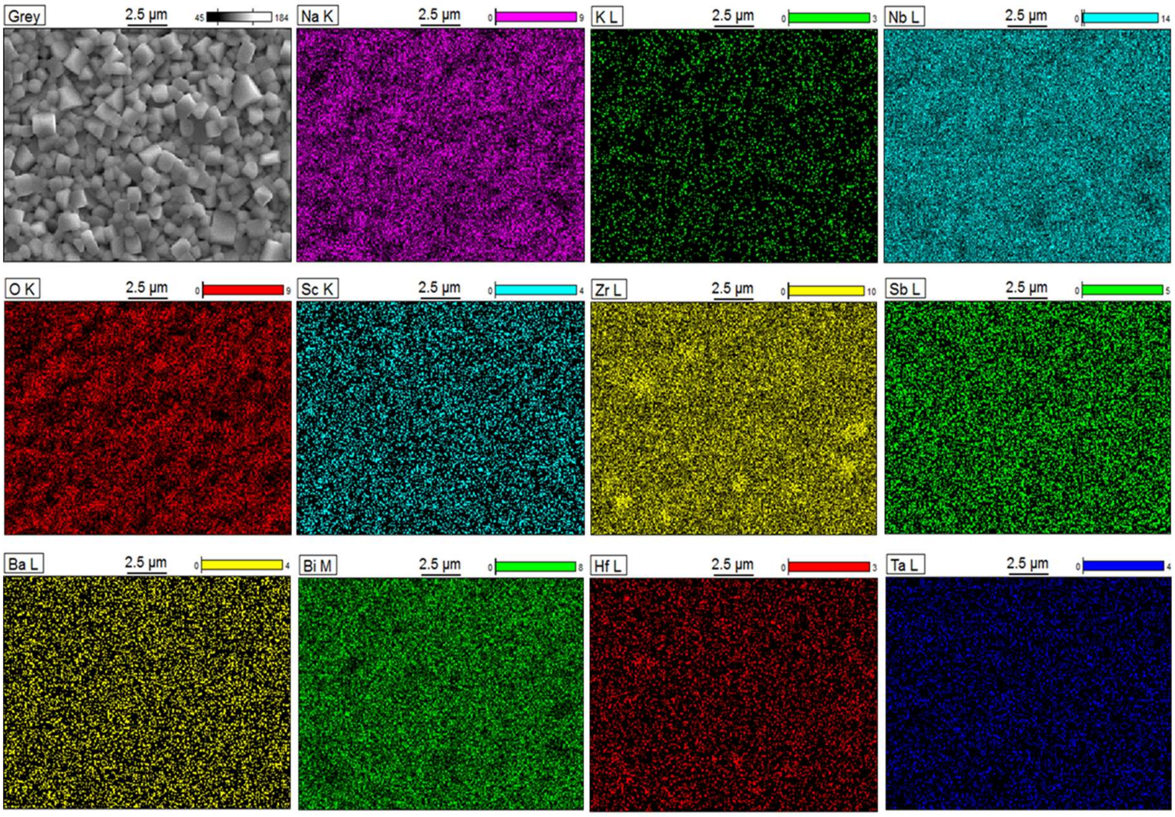

Supplementary Fig. 12 | The element distribution maps of SEM for thermally etched KNN-H ceramic. In KNN-H ceramic, except for Li, which cannot be detected due to its lightness, other elements show a relatively uniform distribution.

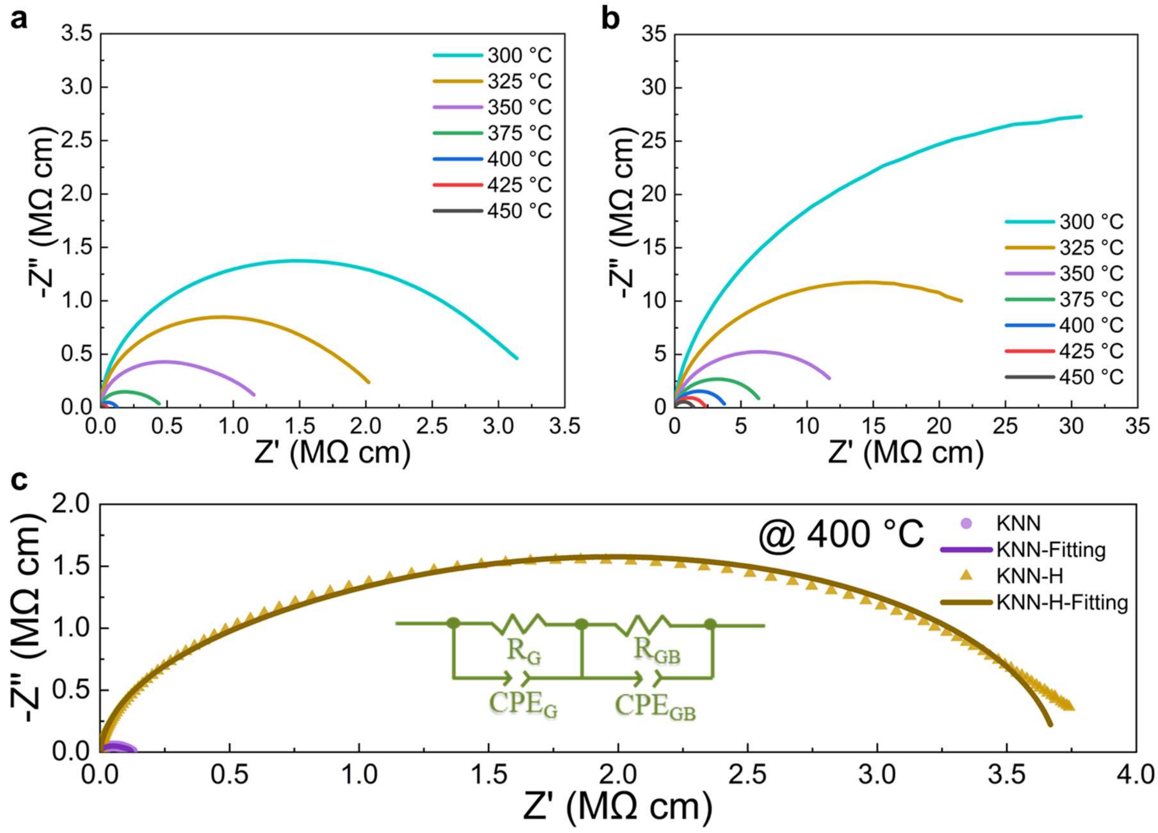

Supplementary Fig. 13 | The impedance performance of KNN and KNN-H ceramics. The Nyquist plots at various temperature for **a** KNN and **b** KNN-H ceramics. **c** The comparison of impedance performance between KNN and KNN-H ceramics at 400 °C. The impedance performance of the studied samples is measured from 300 to 450 °C and in the frequency range of 50 Hz to 2 MHz. As the test temperature increases, the arc curves gradually become smaller, indicating a decrease of the relaxation time of the samples. Furthermore, the resistance value at 400 °C of KNN-H is much larger than that of KNN, leading to a huge improvement of E_b. According to the fitting results by using a series R||CPE equivalent circuit model, it can be seen that all samples show nearly a single semicircle arc, indicating that grain boundary plays a dominant role in the whole conduction.

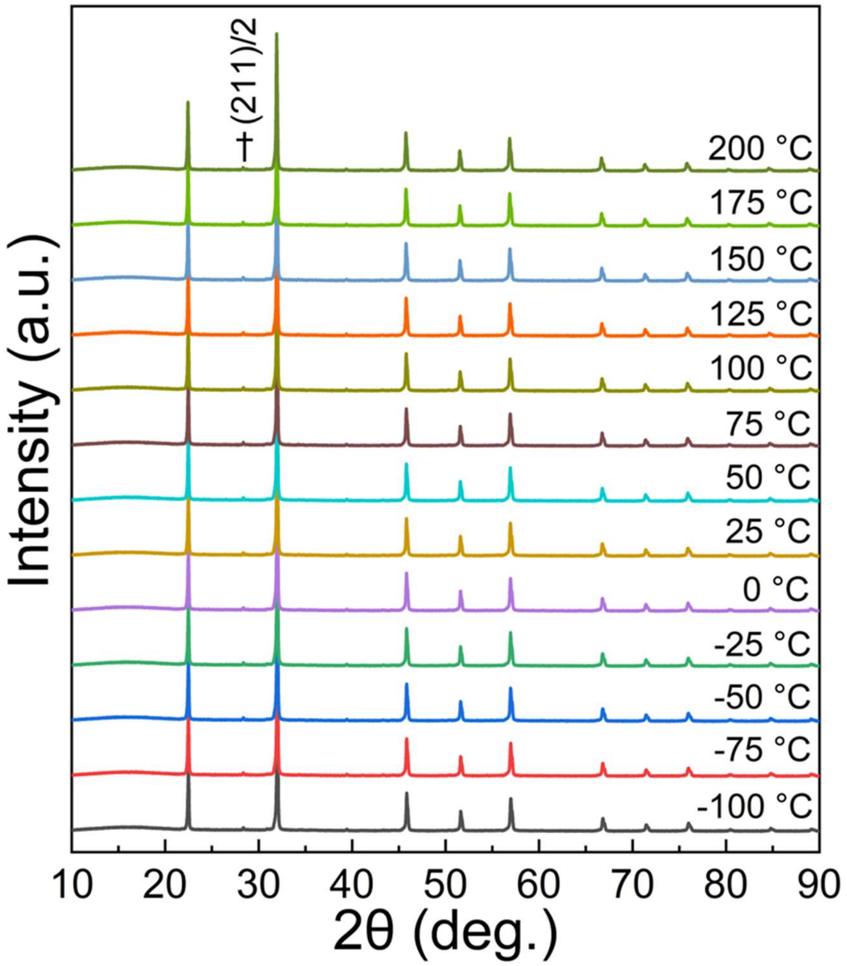

Supplementary Fig. 14 | Temperature-dependent XRD of KNN-H ceramic. "†" represents the positions of the superlattice peaks. KNN-H ceramic exhibits a pseudo-cubic phase structure with almost no change in peak position and number from -100 to 200 °C, indicating excellent temperature-independent structural stability. Notely, the characteristic peak at about 28° representing (211)/2 superlattice always exists, indicating the stable oxygen octahedral distortion. Other superlattice characteristic peaks are not detected due to the limited resolution of XRD. In addition, the pseudocubic perovskite structure indicates that the ions introdued by the high-entropy strategy have diffused into the KNN lattices and a new solid solution of the KNN-H ceramic is formed, which is also responsible for the slim *P*-*E* loops and excellent energy storage efficiency.

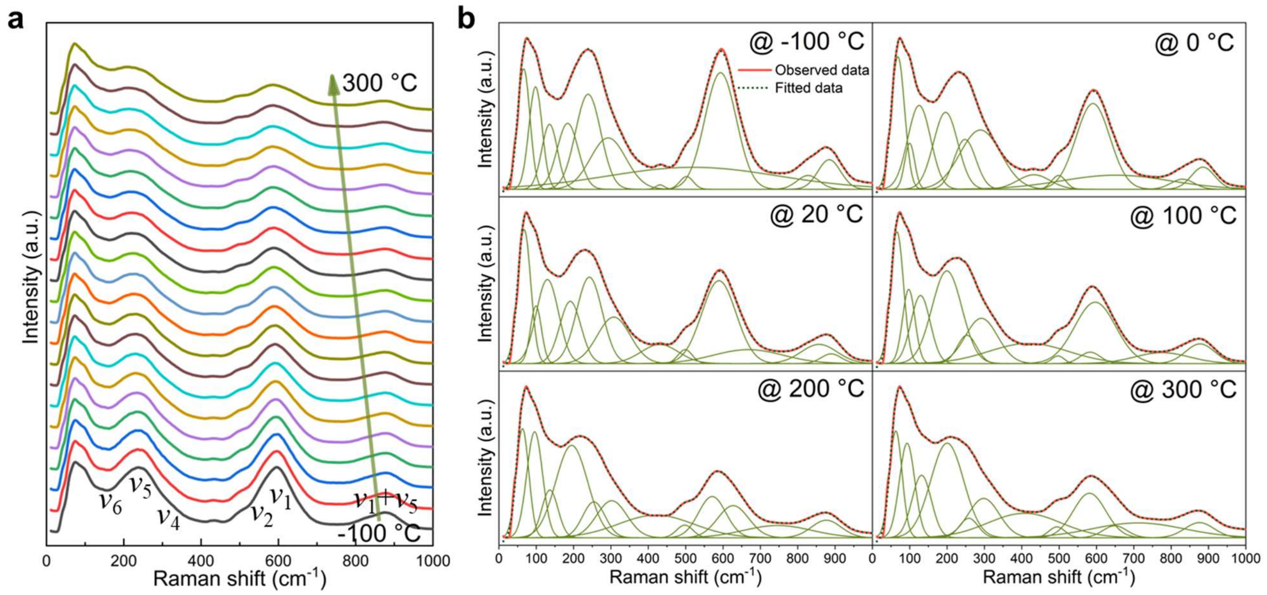

Supplementary Fig. 15 | Temperature-dependent Raman spectra of KNN-H ceramic. a Temperature-dependent Raman spectra from -100 to 300 °C. b The fitted spectra of some selected Raman spectra. It is obvious that broadened and smoothed Raman peaks can be observed with temperature increased from -100 to 300 °C. Yet the number of observed Raman peaks are almost unchanged according to the fitted results, suggesting that nearly no change in local structure symmetry within a wide temperature range. Five relatively strong scattering peaks v_1, v_2, v_4, v_5, and v_6 can be detected, which are assigned to stretching, stretching, bending, bending, and bending vibration modes of the BO_6 octahedron, respectively.[6] The Raman peaks of v_1 and v_5 at around 600 and 240 cm^{-1} shift to the lower frequency on heating, indicating the weakening of the B-O bonds and the hybridization between the Nb^{5+} *d*-orbitals and O *p*-orbitals, which can enhance the local structural disorder and the dynamics of PNRs. The strengthened activity of PNRs can reduce the potential barrier of switching with the electric field and maintain temperature-insensitive high η for energy storage and charge/discharge performance.

Supplementary Table 1

Relevant references for the summary figures.

Figure	*Ref.*
Fig. 4c	7-47
Fig. 4d	7-186
Fig. 5b	186-192
Fig. 5c	94,141,150,193,194

Supplementary Table 2

The values of the Vickers hardness tested in KNN ceramic.

Number	Load /(N)	d_1 /(μm)	d_2 /(μm)	d_{ave} /(μm)	H_v /(GPa)
1	4.9033	52.60	52.80	52.70	3.274
2	4.9033	52.30	50.60	51.45	3.435
3	4.9033	55.00	54.80	54.90	3.017
**Average**				**53.02**	**3.24**

Supplementary Table 3

The values of the Vickers hardness tested in KNN-H ceramic.

Number	Load /(N)	d_1 /(μm)	d_2 /(μm)	d_{ave} /(μm)	H_v /(GPa)
1	4.9033	34.40	34.53	34.465	7.655
2	4.9033	34.64	34.52	34.580	7.604
3	4.9033	34.16	34.03	34.095	7.822
**Average**				**34.38**	**7.70**

Supplementary Table 4

A comparison of the charge/discharge performance between KNN-H ceramic and some recently reported lead-free ceramics.

Compositions	P_D (MW cm^{-3})	W_D (J cm^{-3})	$t_{0.9}$ (ns)	E (kV cm^{-1})	*Ref.*
$0.57BiFeO_3$-$0.33BaTiO_3$-$0.1NaNbO_3$	—	2.5	100	200	45
$Sr_{0.35}Bi_{0.35}K_{0.25}TiO_3$	39.6	1.51	330	200	92
$0.94(Bi_{0.5}K_{0.5})TiO_3$–$0.06La(Mg_{0.5}Ti_{0.5})O_3$	—	0.76	200	140	89
$0.85(0.94(Bi_{0.5}Na_{0.5})TiO_3$-$0.06BaTiO_3)$-$0.15Bi(Mg_{2/3}Nb_{1/3})O_3$	131.4	—	300	350	195
$0.94(Bi_{0.5}Na_{0.5})_{0.65}(Ba_{0.3}Sr_{0.7})_{0.35}TiO_3$-$0.06Bi(Zn_{2/3}Nb_{1/3})O_3$	18.31	0.28	109.2	80	196
$0.75(Bi_{0.58}Na_{0.42})TiO_3$-$0.25SrTiO_3$	147	1.8	118	300	42
$0.8(Bi_{0.5}Na_{0.5})TiO_3$-$0.2Sr(Nb_{0.5}Al_{0.5})O_3$	131.75	—	—	300	197

0.9(0.76($Bi_{0.5}Na_{0.5}$)TiO_3-0.24$SrTiO_3$)-0.1Bi($Ni_{2/3}Nb_{1/3}$)O_3	49.8	0.51	62.4	120	114
0.75($Bi_{0.4465}Na_{0.4465}Ba_{0.057}La_{0.05}$)$TiO_3$-0.25($Sr_{0.85}Bi_{0.1}TiO_3$)	38.1	1.1	45	120	198
0.65$BaTiO_3$-0.35(0.9($Sr_{0.7}Bi_{0.2}$)TiO_3-0.1Bi($Mg_{0.5}Zr_{0.5}$)O_3)	62.6	0.98	62	160	36
0.975($K_{0.5}Na_{0.5}$)NbO_3-0.025$LaBiO_3$	49.4	0.42	46	100	199
0.90($K_{0.5}Na_{0.5}$)NbO_3-0.10Bi($Zn_{2/3}$($Nb_{0.85}Ta_{0.15}$)$_{1/3}$)O_3	184.84	1.81	46.3	400	149
0.9$NaNbO_3$-0.1Bi($Ni_{1/2}Sn_{1/2}$)O_3	100.5	1.11	46.5	200	170
0.92$NaNbO_3$–0.08Bi($Mg_{0.5}Ti_{0.5}$)O_3 -Mn	63.7	1.17	85	200	166
0.62$Sr_{0.7}Bi_{0.2}TiO_3$-0.38($Bi_{0.5}K_{0.5}$)TiO_3	49.5	1.81	360	220	200
0.6($Bi_{0.5}K_{0.5}$)TiO_3-0.3$BaTiO_3$-0.1$NaNbO_3$	103.2	2.4	130	220	94
**KNN-H**	**327.9**	**3.26**	**34.2**	**300**	**This work**

Supplementary References

1 Bokov, A. & Ye, Z.-G. Recent progress in relaxor ferroelectrics with perovskite structure. *J. Mater. Sci.* **41**, 31-52 (2006).

2 Uchino, K. & Nomura, S. Critical exponents of the dielectric constants in diffused-phase-transition crystals. *Ferroelectrics* **44**, 55-61 (1982).

3 Liu, Q. *et al.* High-performance lead-free piezoelectrics with local structural heterogeneity. *Energy Environ. Sci.* **11**, 3531-3539 (2018).

4 Sarkar, A. *et al.* Rare earth and transition metal based entropy stabilised perovskite type oxides. *J. Eur. Ceram. Soc.* **38**, 2318-2327 (2018).

5 Gong, J., Wu, J. & Guan, Z. Examination of the indentation size effect in low-load Vickers hardness testing of ceramics. *J. Eur. Ceram. Soc.* **19**, 2625-2631 (1999).

6 Kakimoto, K.-i., Akao, K., Guo, Y. & Ohsato, H. Raman scattering study of piezoelectric $(Na_{0.5}K_{0.5})NbO_3$-$LiNbO_3$ ceramics. *Jpn. J. Appl. Phys.* **44**, 7064-7067 (2005).

7 Li, L. *et al.* Preparation and dielectric properties of $BaCu(B_2O_5)$-doped $SrTiO_3$-based ceramics for energy storage. *Mater. Sci. Eng. B* **178**, 1509-1514 (2013).

8 Sun, Z., Li, L., Yu, S., Kang, X. & Chen, S. Energy storage properties and relaxor behavior of lead-free $Ba_{1-x}Sm_{2x/3}Zr_{0.15}Ti_{0.85}O_3$ ceramics. *Dalton Trans.* **46**, 14341-14347 (2017).

9 Jiang, X. *et al.* Enhanced energy storage and fast discharge properties of $BaTiO_3$ based ceramics modified by Bi $(Mg_{1/2}Zr_{1/2})O_3$. *J. Eur. Ceram. Soc.* **39**, 1103-1109 (2019).

10 Yang, H. *et al.* A lead free relaxation and high energy storage efficiency ceramics for energy storage applications. *J. Alloys Compd.* **710**, 436-445 (2017).

11 Wang, J., Fan, H., Hu, B. & Jiang, H. Enhanced energy-storage performance and temperature-stable dielectric properties of $(1-x)(0.94Na_{0.5}Bi_{0.5}TiO_3–0.06BaTiO_3)–xNa_{0.73}Bi_{0.09}NbO_3$ ceramics. *Journal of Materials Science: Materials in Electronics* **30**, 2479-2488 (2019).

12 Zhu, C., Cai, Z., Li, L. & Wang, X. High energy density, high efficiency and excellent temperature stability of lead free Mn–doped $BaTiO_3$–Bi $(Mg_{1/2}Zr_{1/2})O_3$ ceramics sintered in a reducing atmosphere. *J. Alloys Compd.* **816**, 152498 (2020).

13 Zhang, L., Pang, L.-X., Li, W.-B. & Zhou, D. Extreme high energy storage efficiency in perovskite structured $(1-x)(Ba_{0.8}Sr_{0.2})TiO_3-xBi(Zn_{2/3}Nb_{1/3})O_3$ $(0.04\leq x\leq 0.16)$ ceramics. *J. Eur. Ceram. Soc.* **40**, 3343-3347 (2020).

14 Li, W.-B., Zhou, D. & Pang, L.-X. Enhanced energy storage density by inducing defect dipoles in lead free relaxor ferroelectric $BaTiO_3$-based ceramics. *Appl. Phys. Lett.* **110**, 132902 (2017).

15 Yang, H., Yan, F., Lin, Y. & Wang, T. Enhanced energy storage properties of $Ba_{0.4}Sr_{0.6}TiO_3$ lead-free ceramics with Bi_2O_3-B_2O_3-SiO_2 glass addition. *J. Eur. Ceram. Soc.* **38**, 1367-1373 (2018).

16 Dai, Z. *et al.* Enhanced energy storage properties and stability of $Sr(Sc_{0.5}Nb_{0.5})O_3$ modified $0.65BaTiO_3$-$0.35Bi_{0.5}Na_{0.5}TiO_3$ ceramics. *Chem. Eng. J.* **397**, 125520 (2020).

17 Huang, Y., Zhao, C., Wu, B. & Wu, J. Multifunctional $BaTiO_3$-based relaxor ferroelectrics toward excellent energy storage performance and electrostrictive strain benefiting from crossover region. *ACS Appl. Mater. Interfaces* **12**, 23885-23895 (2020).

18 Zhou, M., Liang, R., Zhou, Z. & Dong, X. Combining high energy efficiency and fast charge-discharge capability in novel $BaTiO_3$-based relaxor ferroelectric ceramic for energy-storage. *Ceram. Int.* **45**, 3582-3590 (2019).

19 Wang, H. *et al.* Enhanced energy density and discharged efficiency of lead-free relaxor $(1-x)[(Bi_{0.5}Na_{0.5})_{0.94}Ba_{0.06}]_{0.98}La_{0.02}TiO_3-xKNb_{0.6}Ta_{0.4}O_3$ ceramic capacitors. *Chem. Eng. J.* **394**, 124879 (2020).

20 Zhao, P. *et al.* Novel Ca doped $Sr_{0.7}Bi_{0.2}TiO_3$ lead-free relaxor ferroelectrics with high energy density and efficiency. *J. Eur. Ceram. Soc.* **40**, 1938-1946 (2020).

21 Zhou, M., Liang, R., Zhou, Z. & Dong, X. Novel $BaTiO_3$-based lead-free ceramic capacitors featuring high energy storage density, high power density, and excellent stability. *J. Mater. Chem. C* **6**, 8528-8537 (2018).

22 Si, F., Tang, B., Fang, Z., Li, H. & Zhang, S. A new type of $BaTiO_3$-based ceramics with Bi $(Mg_{1/2}Sn_{1/2})O_3$ modification showing improved energy storage properties and pulsed discharging performances. *J. Alloys Compd.* **819**, 153004 (2020).

23 Chen, Z. *et al.* Simultaneously achieving high energy storage density and efficiency under low electric field in $BiFeO_3$-based lead-free relaxor ferroelectric ceramics. *J. Eur. Ceram. Soc.* **40**, 5450-5457 (2020).

24 Wang, Q., Gong, P.-M. & Wang, C.-M. High recoverable energy storage density and large energy efficiency simultaneously achieved in $BaTiO_3$–$Bi(Zn_{1/2}Zr_{1/2})O_3$ relaxor ferroelectrics. *Ceram. Int.* **46**, 22452-22459 (2020).

25 Chen, X. *et al.* Achieving ultrahigh energy storage density and energy efficiency simultaneously in barium titanate based ceramics. *Appl. Phys. A* **126**, 1-8 (2020).

26 Si, F., Tang, B., Fang, Z., Li, H. & Zhang, S. Enhanced energy storage and fast charge-discharge properties of $(1\text{-}x)BaTiO_3\text{-}xBi(Ni_{1/2}Sn_{1/2})O_3$ relaxor ferroelectric ceramics. *Ceram. Int.* **45**, 17580-17590 (2019).

27 Kang, R. *et al.* Domain engineered lead-free ceramics with large energy storage density and ultra-high efficiency under low electric fields. *ACS Appl. Mater. Interfaces* **13**, 25143-25152 (2021).

28 Li, X., Chen, X., Sun, J., Zhou, M. & Zhou, H. Novel lead-free ceramic capacitors with high energy density and fast discharge performance. *Ceram. Int.* **46**, 3426-3432 (2020).

29 Kong, X., Yang, L., Cheng, Z. & Zhang, S. Bi-modified $SrTiO_3$-based ceramics for high-temperature energy storage applications. *J. Am. Ceram. Soc.* **103**, 1722-1731 (2020).

30 Huang, Y., Guo, Q., Hao, H., Liu, H. & Zhang, S. Tailoring properties of $(Bi_{0.51}Na_{0.47})$ TiO_3 based dielectrics for energy storage applications. *J. Eur. Ceram. Soc.* **39**, 4752-4760 (2019).

31 Wang, Y. *et al.* Structure, dielectric properties of novel $Ba(Zr,Ti)O_3$ based ceramics for energy storage application. *Ceram. Int.* **46**, 12080-12087 (2020).

32 Pu, Y. *et al.* Enhancing the energy storage properties of $Ca_{0.5}Sr_{0.5}TiO_3$-based lead-free linear dielectric ceramics with excellent stability through regulating grain boundary defects. *J. Mater. Chem. C* **7**, 14384-14393 (2019).

33 Yang, L., Kong, X., Cheng, Z. & Zhang, S. Ultra-high energy storage performance with mitigated polarization saturation in lead-free relaxors. *J. Mater. Chem. A* **7**, 8573-8580 (2019).

34 Zhou, M., Liang, R., Zhou, Z. & Dong, X. Achieving ultrahigh energy storage density and energy efficiency simultaneously in sodium niobate-based lead-free dielectric capacitors via microstructure modulation. *Inorg. Chem. Front.* **6**, 2148-2157 (2019).

35 Zhang, X. *et al.* Enhancement of recoverable energy density and efficiency of lead-free relaxor-ferroelectric BNT-based ceramics. *Chem. Eng. J.* **406**, 126818 (2021).

36 Hu, D. *et al.* Optimization the energy density and efficiency of $BaTiO_3$-based ceramics for capacitor applications. *Chem. Eng. J.* **409**, 127375 (2021).

37 Qiao, X. *et al.* Superior comprehensive energy storage properties in $Bi_{0.5}Na_{0.5}TiO_3$-based relaxor ferroelectric ceramics. *Chem. Eng. J.* **388**, 124158 (2020).

38 Dong, X., Li, X., Chen, X., Wu, J. & Zhou, H. Simultaneous enhancement of polarization and breakdown strength in lead-free $BaTiO_3$-based ceramics. *Chem. Eng. J.* **409**, 128231 (2021).

39 Hu, Q. *et al.* Achieve ultrahigh energy storage performance in $BaTiO_3$–$Bi(Mg_{1/2}Ti_{1/2})O_3$ relaxor ferroelectric ceramics via nano-scale polarization mismatch and reconstruction. *Nano Energy* **67**, 104264 (2020).

40 Wei, T. *et al.* Novel $NaNbO_3$–$Sr_{0.7}Bi_{0.2}TiO_3$ lead-free dielectric ceramics with excellent energy storage properties. *Ceram. Int.* **47**, 3713-3719 (2021).

41 Yang, H. *et al.* Novel $BaTiO_3$-based, Ag/Pd-compatible lead-free relaxors with superior energy storage performance. *ACS Appl. Mater. Interfaces* **12**, 43942-43949 (2020).

42 Yan, F. *et al.* Significantly enhanced energy storage density and efficiency of BNT-based perovskite ceramics via A-site defect engineering. *Energy Storage Mater.* **30**, 392-400 (2020).

43 Ji, H. *et al.* Ultrahigh energy density in short-range tilted NBT-based lead-free multilayer ceramic capacitors by nanodomain percolation. *Energy Storage Mater.* **38**, 113-120 (2021).

44 Chen, H. *et al.* Excellent energy storage properties and stability of $NaNbO_3$–$Bi(Mg_{0.5}Ta_{0.5})O_3$ ceramics by introducing $(Bi_{0.5}Na_{0.5})_{0.7}Sr_{0.3}TiO_3$. *J. Mater. Chem. A* **9**, 4789-4799 (2021).

45 Qi, H., Xie, A., Tian, A. & Zuo, R. Superior energy-storage capacitors with simultaneously giant energy density and efficiency using nanodomain engineered $BiFeO_3$-$BaTiO_3$-$NaNbO_3$ lead‐free bulk ferroelectrics. *Adv. Energy Mater.* **10**, 1903338 (2020).

46 Li, D., Zhou, D., Wang, D., Zhao, W., Guo, Y. & Shi, Z. Improved energy storage properties achieved in $(K,Na)NbO_3$-based relaxor ferroelectric ceramics via a combinatorial optimization strategy. *Adv. Funct. Mater.* 2022, **32**, 2111776.

47 Luo, N. *et al.* Constructing phase boundary in $AgNbO_3$ antiferroelectrics: pathway simultaneously achieving high energy density and efficiency. *Nat. Commun.* **11**, 1-10 (2020).

48 Luo, N. *et al.* Aliovalent A-site engineered $AgNbO_3$ lead-free antiferroelectric ceramics toward superior energy storage density. *J. Mater. Chem. A* **7**, 14118-14128 (2019).

49 Gao, J. *et al.* Antiferroelectric-ferroelectric phase transition in lead-free $AgNbO_3$ ceramics for energy storage applications. *J. Am. Ceram. Soc.* **101**, 5443-5450 (2018).

50 Mao, S. *et al.* Effect of Lu doping on the structure, electrical properties and energy storage performance of $AgNbO_3$ antiferroelectric ceramics. *Journal of Materials Science: Materials in Electronics* **31**, 7731-7741 (2020).

51 Song, A. *et al.* Energy storage performance in $BiMnO_3$-modified $AgNbO_3$ anti-ferroelectric ceramics. *Mater. Lett.* **237**, 278-281 (2019).

52 Gao, J. *et al.* Enhanced antiferroelectric phase stability in La-doped $AgNbO_3$: perspectives from the microstructure to energy storage properties. *J. Mater. Chem. A* **7**, 2225-2232 (2019).

53 Ren, P. *et al.* Grain size tailoring and enhanced energy storage properties of two-step sintered Nd^{3+}-doped $AgNbO_3$. *J. Eur. Ceram. Soc.* **40**, 4495-4502 (2020).

54 Tian, Y. *et al.* High energy density in silver niobate ceramics. *J. Mater. Chem. A* **4**, 17279-17287 (2016).

55 Xu, Y. *et al.* High energy storage properties of lead-free Mn-doped $(1-x)AgNbO_3$-$xBi_{0.5}Na_{0.5}TiO_3$ antiferroelectric ceramics. *J. Eur. Ceram. Soc.* **40**, 56-62 (2020).

56 Xu, C. *et al.* La/Mn codoped $AgNbO_3$ lead-free antiferroelectric ceramics with large energy density and power density. *ACS Sustainable Chem. Eng.* **6**, 16151-16159 (2018).

57 Luo, N. *et al.* Lead-free $Ag_{1-3x}La_xNbO_3$ antiferroelectric ceramics with high-energy storage density and efficiency. *J. Am. Ceram. Soc.* **102**, 4640-4647 (2019).

58 Zhao, L., Liu, Q., Zhang, S. & Li, J.-F. Lead-free $AgNbO_3$ anti-ferroelectric ceramics with an enhanced energy storage performance using MnO_2 modification. *J. Mater. Chem. C* **4**, 8380-8384 (2016).

59 Zhao, L., Liu, Q., Gao, J., Zhang, S. & Li, J. F. Lead-free antiferroelectric silver niobate tantalate with high energy storage performance. *Adv. Mater.* **29**, 1701824 (2017).

60 Gao, J. *et al.* Local structure heterogeneity in Sm-doped $AgNbO_3$ for improved energy-storage performance. *ACS Appl. Mater. Interfaces* **12**, 6097-6104 (2020).

61 Lu, Z. *et al.* Mechanism of enhanced energy storage density in $AgNbO_3$-based lead-free antiferroelectrics. *Nano Energy* **79**, 105423 (2021).

62 Tian, Y. *et al.* Phase transitions in bismuth-modified silver niobate ceramics for high power energy storage. *J. Mater. Chem. A* **5**, 17525-17531 (2017).
63 Han, K. *et al.* Realizing high low-electric-field energy storage performance in $AgNbO_3$ ceramics by introducing relaxor behaviour. *J. Materiomics* **5**, 597-605 (2019).
64 Li, S. *et al.* Significantly enhanced energy storage performance of rare-earth-modified silver niobate lead-free antiferroelectric ceramics via local chemical pressure tailoring. *J. Mater. Chem. C* **7**, 1551-1560 (2019).
65 Yan, Z. *et al.* Silver niobate based lead-free ceramics with high energy storage density. *J. Mater. Chem. A* **7**, 10702-10711 (2019).
66 Zhao, L., Gao, J., Liu, Q., Zhang, S. & Li, J.-F. Silver niobate lead-free antiferroelectric ceramics: enhancing energy storage density by B-site doping. *ACS Appl. Mater. Interfaces* **10**, 819-826 (2018).
67 Han, K. *et al.* Ultrahigh energy-storage density in A-/B-site co-doped $AgNbO_3$ lead-free antiferroelectric ceramics: insight into the origin of antiferroelectricity. *J. Mater. Chem. A* **7**, 26293-26301 (2019).
68 Li, S. *et al.* Giant energy density and high efficiency achieved in silver niobate-based lead-free antiferroelectric ceramic capacitors via domain engineering. *Energy Storage Mater.* **34**, 417-426 (2021).
69 Chao, W., Yang, T., Li, Y. & Liu, Z. Enhanced energy storage density in Ca and Ta co-doped $AgNbO_3$ antiferroelectric ceramics. *J. Am. Ceram. Soc.* **103**, 7283-7290 (2020).
70 Chao, W., Gao, J., Yang, T. & Li, Y. Excellent energy storage performance in La and Ta co-doped $AgNbO_3$ antiferroelectric ceramics. *J. Eur. Ceram. Soc.* **41**, 7670-7677 (2021).
71 Li, J. *et al.* Silver deficiency effect on dielectric properties and energy storage performance of $AgNbO_3$ ceramics. *Ceram. Int.* **47**, 26178-26184 (2021).
72 Shi, P. *et al.* Significantly enhanced energy storage properties of Nd^{3+} doped $AgNbO_3$ lead-free antiferroelectric ceramics. *J. Alloys Compd.* **877**, 160162 (2021).
73 Luo, N. *et al.* Silver stoichiometry engineering: an alternative way to improve energy storage density of $AgNbO_3$-based antiferroelectric ceramics. *J. Mater. Res.* **36**, 1067-1075 (2021).
74 Xu, Y. *et al.* Enhanced energy density in Mn-doped $(1-x)AgNbO_3$-$x CaTiO_3$ lead-free antiferroelectric ceramics. *J. Alloys Compd.* **821**, 153260 (2020).
75 Liu, N., Liang, R., Zhou, Z. & Dong, X. Designing lead-free bismuth ferrite-based ceramics learning from relaxor ferroelectric behavior for simultaneous high energy density and efficiency under low electric field. *J. Mater. Chem. C* **6**, 10211-10217 (2018).
76 Zheng, D. & Zuo, R. Enhanced energy storage properties in $La(Mg_{1/2}Ti_{1/2})O_3$-modified $BiFeO_3$-$BaTiO_3$ lead-free relaxor ferroelectric ceramics within a wide temperature range. *J. Eur. Ceram. Soc.* **37**, 413-418 (2017).
77 Dabas, S., Kumar, M., Chaudhary, P. & Thakur, O. Enhanced magneto-electric coupling and energy storage analysis in Mn-modified lead free $BiFeO_3$-$BaTiO_3$ solid solutions. *J. Appl. Phys.* **126**, 134102 (2019).
78 Wang, D. *et al.* High energy storage density and large strain in $Bi(Zn_{2/3}Nb_{1/3})O_3$-doped $BiFeO_3$–$BaTiO_3$ ceramics. *ACS Appl. Energy Mater.* **1**, 4403-4412 (2018).
79 Akram, F. *et al.* Less temperature-dependent high dielectric and energy-storage properties of eco-friendly $BiFeO_3$–$BaTiO_3$-based ceramics. *J. Alloys Compd.* **818**, 152878 (2020).
80 Zheng, D., Zuo, R., Zhang, D. & Li, Y. Novel $BiFeO_3$‑BaTiO3‑$Ba(Mg_{1/3}Nb_{2/3})O_3$ lead-free relaxor ferroelectric ceramics for energy-storage capacitors. *J. Am. Ceram. Soc.* **98**, 2692-2695 (2015).
81 Liu, N. *et al.* Novel bismuth ferrite-based lead-free ceramics with high energy and power density. *J. Am. Ceram. Soc.* **101**, 3259-3265 (2018).
82 Lu, Z. *et al.* Superior energy density through tailored dopant strategies in multilayer ceramic capacitors. *Energy Environ. Sci.* **13**, 2938-2948 (2020).
83 Chen, Z. *et al.* Achieving high-energy storage performance in $0.67Bi_{1-x}Sm_xFeO_3$-$0.33BaTiO_3$ lead-free relaxor ferroelectric ceramics. *Ceram. Int.* **46**, 11549-11555 (2020).

84 Yang, H., Qi, H. & Zuo, R. Enhanced breakdown strength and energy storage density in a new $BiFeO_3$-based ternary lead-free relaxor ferroelectric ceramic. *J. Eur. Ceram. Soc.* **39**, 2673-2679 (2019).
85 Wang, G. *et al.* Fatigue resistant lead-free multilayer ceramic capacitors with ultrahigh energy density. *J. Mater. Chem. A* **8**, 11414-11423 (2020).
86 Sun, H. *et al.* Large energy storage density in $BiFeO_3$-$BaTiO_3$-$AgNbO_3$ lead-free relaxor ceramics. *J. Eur. Ceram. Soc.* **40**, 2929-2935 (2020).
87 Bai, X. *et al.* High recoverable energy storage density in nominal (0.67-x) $BiFeO_3$-0.33$BaTiO_3$-x$BaBi_2Nb_2O_9$ lead-free composite ceramics. *Ceram. Int.* **47**, 23116-23123 (2021).
88 Shiga, M., Hagiwara, M. & Fujihara, S. $(Bi_{1/2}K_{1/2})TiO_3$–$SrTiO_3$ solid-solution ceramics for high-temperature capacitor applications. *Ceram. Int.* **46**, 10242-10249 (2020).
89 Li, F., Jiang, T., Zhai, J., Shen, B. & Zeng, H. Exploring novel bismuth-based materials for energy storage applications. *J. Mater. Chem. C* **6**, 7976-7981 (2018).
90 Li, F. *et al.* Fine-grain induced outstanding energy storage performance in novel $Bi_{0.5}K_{0.5}TiO_3$–$Ba(Mg_{1/3}Nb_{2/3})O_3$ ceramics via a hot-pressing strategy. *J. Mater. Chem. C* **7**, 12127-12138 (2019).
91 Li, F., Si, R., Li, T., Wang, C. & Zhai, J. High energy storage performance and fast discharging speed in dense 0.7$Bi_{0.5}K_{0.5}TiO_3$-0.3$SrTiO_3$ ceramics via a novel rolling technology. *Ceram. Int.* **46**, 6995-6998 (2020).
92 Zhao, P. *et al.* Improved dielectric breakdown strength and energy storage properties in Er_2O_3 modified $Sr_{0.35}Bi_{0.35}K_{0.25}TiO_3$. *Chem. Eng. J.* **403**, 126290 (2021).
93 Yang, Q. *et al.* Excellent energy storage performance of $K_{0.5}Bi_{0.5}TiO_3$-based ferroelectric ceramics under low electric field. *Chem. Eng. J.* **414**, 128769 (2021).
94 Chen, L. *et al.* Outstanding Energy Storage Performance in High-Hardness $(Bi_{0.5}K_{0.5})TiO_3$-Based Lead-Free Relaxors via Multi-Scale Synergistic Design. *Adv. Funct. Mater.* **32**, 2110478 (2022).
95 Hu, D. *et al.* Greatly enhanced discharge energy density and efficiency of novel relaxation ferroelectric BNT–BKT-based ceramics. *J. Mater. Chem. C* **8**, 591-601 (2020).
96 Li, T. *et al.* High energy storage density and efficiency with excellent temperature and frequency stabilities under low operating field achieved in $Ag_{0.91}Sm_{0.03}NbO_3$-modified $Na_{0.5}Bi_{0.5}TiO_3$-$BaTiO_3$ ceramics. *Journal of Materials Science: Materials in Electronics* **31**, 16928-16937 (2020).
97 Zhang, F. *et al.* High energy storage density realized in $Bi_{0.5}Na_{0.5}TiO_3$-based relaxor ferroelectric ceramics at ultralow sintering temperature. *J. Eur. Ceram. Soc.* **41**, 368-375 (2021).
98 Hu, B., Fan, H., Ning, L., Wen, Y. & Wang, C. High energy storage performance of $[(Bi_{0.5}Na_{0.5})_{0.94}Ba_{0.06}]_{0.97}La_{0.03}Ti_{1-x}(Al_{0.5}Nb0_{.5})_xO_3$ ceramics with enhanced dielectric breakdown strength. *Ceram. Int.* **44**, 15160-15166 (2018).
99 Zhu, C. *et al.* High temperature lead-free BNT-based ceramics with stable energy storage and dielectric properties. *J. Mater. Chem. A* **8**, 683-692 (2020).
100 Zhang, L., Pu, X., Chen, M., Bai, S. & Pu, Y. Influence of BaSnO3 additive on the energy storage properties of $Na_{0.5}Bi_{0.5}TiO_3$-based relaxor ferroelectrics. *J. Eur. Ceram. Soc.* **38**, 2304-2311 (2018).
101 Zhang, L., Pu, Y. & Chen, M. Influence of $BaZrO_3$ additive on the energy-storage properties of 0.775$Na_{0.5}Bi_{0.5}TiO_3$-0.225$BaSnO_3$ relaxor ferroelectrics. *J. Alloys Compd.* **775**, 342-347 (2019).
102 Liu, Z. *et al.* Mediating the confliction of polarizability and breakdown electric-field strength in BNST relaxor ferroelectric for energy storage applications. *J. Alloys Compd.* **823**, 153772 (2020).
103 Wu, J. *et al.* Perovskite $Sr_x(Bi_{1-x}Na_{0.97-x}Li_{0.03})_{0.5}TiO_3$ ceramics with polar nano regions for high power energy storage. *Nano Energy* **50**, 723-732 (2018).
104 Hu, D. *et al.* Significantly improved recoverable energy density and ultrafast discharge rate of $Na_{0.5}Bi_{0.5}TiO_3$-based ceramics. *Ceram. Int.* **46**, 15364-15371 (2020).

105 Zhang, L., Pu, Y. & Chen, M. Ultra-high energy storage performance under low electric fields in $Na_{0.5}Bi_{0.5}TiO_3$-based relaxor ferroelectrics for pulse capacitor applications. *Ceram. Int.* **46**, 98-105 (2020).

106 Yin, J., Zhang, Y., Lv, X. & Wu, J. Ultrahigh energy-storage potential under low electric field in bismuth sodium titanate-based perovskite ferroelectrics. *J. Mater. Chem. A* **6**, 9823-9832 (2018).

107 Qi, H. & Zuo, R. Linear-like lead-free relaxor antiferroelectric $(Bi_{0.5}Na_{0.5})TiO_3$–$NaNbO_3$ with giant energy-storage density/efficiency and super stability against temperature and frequency. *J. Mater. Chem. A* **7**, 3971-3978 (2019).

108 Zhang, C. *et al.* Superior energy-storage performance in $0.85Bi_{0.5}Na_{0.5}TiO_3$–$0.15NaNbO_3$ lead-free ferroelectric ceramics via composition and microstructure engineering. *J. Mater. Chem. A* **9**, 10088-10094 (2021).

109 Zhou, X. *et al.* Superior thermal stability of high energy density and power density in domain-engineered $Bi_{0.5}Na_{0.5}TiO_3$–$NaTaO_3$ relaxor ferroelectrics. *ACS Appl. Mater. Interfaces* **11**, 43107-43115 (2019).

110 Kang, R. *et al.* Energy storage performance of $Bi_{0.5}Na_{0.5}TiO_3$-based relaxor ferroelectric ceramics with superior temperature stability under low electric fields. *Chem. Eng. J.* **410**, 128376 (2021).

111 Jiang, Z., Yang, Z., Yuan, Y., Tang, B. & Zhang, S. High energy storage properties and dielectric temperature stability of $(1\text{-}x)(0.8Bi_{0.5}Na_{0.5}TiO_3\text{-}0.2Ba_{0.3}Sr_{0.7}TiO_3)\text{-}x NaNbO_3$ lead-free ceramics. *J. Alloys Compd.* **851**, 156821 (2021).

112 He, Z. *et al.* Low electric field induced high energy storage capability of the free-lead relaxor ferroelectric $0.94Bi_{0.5}Na_{0.5}TiO_3$-$0.06BaTiO_3$-based ceramics. *Ceram. Int.* **47**, 11611-11617 (2021).

113 Bilal, M. K., Bashir, R., Asif, S. U., Wang, J. & Hu, W. Enhanced energy storage properties of $0.7Bi_{0.5}Na_{0.5}TiO_3$-$0.3SrTiO_3$ ceramic through the addition of $NaNbO_3$. *Ceram. Int.* **47**, 30922-30928 (2021).

114 Yang, H., Tian, J., Lin, Y. & Ma, J. Realizing ultra-high energy storage density of lead-free $0.76Bi_{0.5}Na_{0.5}TiO_3$-$0.24SrTiO_3$-$Bi(Ni_{2/3}Nb_{1/3})O_3$ ceramics under low electric fields. *Chem. Eng. J.* **418**, 129337 (2021).

115 Shi, P. *et al.* $Bi_{0.5}Na_{0.5}TiO_3$-based lead-free ceramics with superior energy storage properties at high temperatures. *Composites Part B: Engineering* **215**, 108815 (2021).

116 Guo, X., Shi, P., Lou, X., Liu, Q. & Zuo, H. Superior energy storage properties in $(1-x)(0.65Bi_{0.5}Na_{0.5}TiO_3$-$0.35Bi_{0.2}Sr_{0.7}TiO_3)\text{-}x CaZrO_3$ ceramics with excellent temperature stability. *J. Alloys Compd.* **876**, 160101 (2021).

117 Wang, M. *et al.* Relaxor ferroelectric $Bi_{0.5}Na_{0.5}TiO_3$–$Sr_{0.7}Nd_{0.2}TiO_3$ ceramics with high energy storage density and excellent stability under a low electric field. *J. Phys. Chem. Solids* **157**, 110209 (2021).

118 Yao, K. *et al.* $Bi_{0.5}Na_{0.5}TiO_3$–$Sr_{0.85}Bi_{0.1}TiO_3$ ceramics with high energy storage properties and extremely fast discharge speed via regulating relaxation temperature. *Ceram. Int.* **47**, 11294-11303 (2021).

119 Zhu, C. *et al.* Multiphase engineered BNT-based ceramics with simultaneous high polarization and superior breakdown strength for energy storage applications. *ACS Appl. Mater. Interfaces* **13**, 28484-28492 (2021).

120 Zheng, L. *et al.* Significantly tailored energy-storage performances in $Bi_{0.5}Na_{0.5}TiO_3$–$SrTiO_3$-based relaxor ferroelectric ceramics by introducing bismuth layer-structured relaxor $BaBi_2Nb_2O_9$ for capacitor application. *J. Mater. Chem. C* **9**, 5234-5243 (2021).

121 Guo, B. *et al.* Energy storage performance of $Na_{0.5}Bi_{0.5}TiO_3$ based lead-free ferroelectric ceramics prepared via non-uniform phase structure modification and rolling process. *Chem. Eng. J.* **420**, 130475 (2021).

122 Liu, W., Gao, J., Zhao, Y. & Li, S. Significant enhancement of energy storage properties of $BaTiO_3$-based ceramics by hybrid-doping. *J. Alloys Compd.* **843**, 155938 (2020).

123 Yuan, Q. *et al.* Simultaneously achieved temperature-insensitive high energy density and efficiency in domain engineered $BaTiO_3$-$Bi(Mg_{0.5}Zr_{0.5})O_3$ lead-free relaxor ferroelectrics. *Nano Energy* **52**, 203-210 (2018).

124 Li, W.-B., Zhou, D. & Pang, L.-X. Structure and energy storage properties of Mn-doped $(Ba,Sr)TiO_3$–MgO composite ceramics. *Journal of Materials Science: Materials in Electronics* **28**, 8749-8754 (2017).
125 Liu, G. *et al.* An investigation of the dielectric energy storage performance of $Bi(Mg_{2/3}Nb_{1/3})O_3$-modifed $BaTiO_3$ Pb-free bulk ceramics with improved temperature/frequency stability. *Ceram. Int.* **45**, 19189-19196 (2019).
126 Shen, Z., Wang, X., Luo, B. & Li, L. $BaTiO_3$–$BiYbO_3$ perovskite materials for energy storage applications. *J. Mater. Chem. A* **3**, 18146-18153 (2015).
127 Jain, A., Wang, Y., Wang, N. & Wang, F. Critical role of CuO doping on energy storage performance and electromechanical properties of $Ba_{0.8}Sr_{0.1}Ca_{0.1}Ti_{0.9}Zr_{0.1}O_3$ ceramics. *Ceram. Int.* **46**, 18800-18812 (2020).
128 Hu, Q. *et al.* Dielectric and temperature stable energy storage properties of $0.88BaTiO_3$–$0.12Bi(Mg_{1/2}Ti_{1/2})O_3$ bulk ceramics. *J. Alloys Compd.* **640**, 416-420 (2015).
129 Zhan, D. *et al.* Dielectric nonlinearity and electric breakdown behaviors of $Ba_{0.95}Ca_{0.05}Zr_{0.3}Ti_{0.7}O_3$ ceramics for energy storage utilizations. *J. Alloys Compd.* **682**, 594-600 (2016).
130 Li, Y. & Bian, J. Effects of reoxidation on the dielectric and energy storage properties of Ce-doped $(Ba,Sr)TiO_3$ ceramics prepared by hot-pressed sintering. *J. Eur. Ceram. Soc.* **40**, 5441-5449 (2020).
131 Liu, X. *et al.* Enhanced energy storage properties of $BaTiO_3$-$Bi_{0.5}Na_{0.5}TiO_3$ lead-free ceramics modified by $SrY_{0.5}Nb_{0.5}O_3$. *J. Alloys Compd.* **778**, 97-104 (2019).
132 Liu, Z.-G. *et al.* Excellent energy storage density and efficiency in lead-free Sm-doped $BaTiO_3$–$Bi(Mg_{0.5}Ti_{0.5})O_3$ ceramics. *J. Mater. Chem. C* **8**, 13405-13414 (2020).
133 Jain, A., Wang, Y. & Guo, H. Microstructural properties and ultrahigh energy storage density in $Ba_{0.9}Ca_{0.1}TiO_3$–$NaNb_{0.85}Ta_{0.15}O_3$ relaxor ceramics. *Ceram. Int.* **46**, 24333-24346 (2020).
134 Liu, G. *et al.* Ultrahigh dielectric breakdown strength and excellent energy storage performance in lead-free barium titanate-based relaxor ferroelectric ceramics via a combined strategy of composition modification, viscous polymer processing, and liquid-phase sintering. *Chem. Eng. J.* **398**, 125625 (2020).
135 Huang, W. *et al.* Ultrahigh recoverable energy storage density and efficiency in barium strontium titanate-based lead-free relaxor ferroelectric ceramics. *Appl. Phys. Lett.* **113**, 203902 (2018).
136 Dai, Z. *et al.* Effective strategy to achieve excellent energy storage properties in lead-free BaTiO3-based bulk ceramics. *ACS Appl. Mater. Interfaces* **12**, 30289-30296 (2020).
137 Li, Y. *et al.* Energy storage performance of $BaTiO_3$-based relaxor ferroelectric ceramics prepared through a two-step process. *Chem. Eng. J.* **419**, 129673 (2021).
138 Liu, Z.-G., Li, M.-D., Tang, Z.-H. & Tang, X.-G. Enhanced energy storage density and efficiency in lead-free $Bi(Mg_{1/2}Hf_{1/2})O_3$-modified $BaTiO_3$ ceramics. *Chem. Eng. J.* **418**, 129379 (2021).
139 Zhang, M., Yang, H., Li, D. & Lin, Y. Excellent energy density and power density achieved in $K_{0.5}Na_{0.5}NbO_3$-based ceramics with high optical transparency. *J. Alloys Compd.* **829**, 154565 (2020).
140 Zhang, M., Yang, H., Li, D., Ma, L. & Lin, Y. Giant energy storage efficiency and high recoverable energy storage density achieved in $K_{0.5}Na_{0.5}NbO_3$-$Bi(Zn_{0.5}Zr_{0.5})O_3$ ceramics. *J. Mater. Chem. C* **8**, 8777-8785 (2020).
141 Yang, Z. *et al.* Grain size engineered lead-free ceramics with both large energy storage density and ultrahigh mechanical properties. *Nano Energy* **58**, 768-777 (2019).
142 Chai, Q., Yang, D., Zhao, X., Chao, X. & Yang, Z. Lead-free $(K,Na)NbO_3$-based ceramics with high optical transparency and large energy storage ability. *J. Am. Ceram. Soc.* **101**, 2321-2329 (2018).
143 Qu, B., Du, H. & Yang, Z. Lead-free relaxor ferroelectric ceramics with high optical transparency and energy storage ability. *J. Mater. Chem. C* **4**, 1795-1803 (2016).
144 Jia, Q. *et al.* Photochromic and energy storage properties in $K_{0.5}Na_{0.5}NbO_3$-based ferroelectrics. *Journal of Materials Science: Materials in Electronics* **31**, 19277-19292 (2020).

145 Shao, T. *et al.* Potassium–sodium niobate based lead-free ceramics: novel electrical energy storage materials. *J. Mater. Chem. A* **5**, 554-563 (2017).
146 Yang, Z. *et al.* Significantly enhanced recoverable energy storage density in potassium–sodium niobate-based lead free ceramics. *J. Mater. Chem. A* **4**, 13778-13785 (2016).
147 Chen, B. *et al.* Ultrahigh storage density achieved with (1-*x*)KNN-*x*BZN ceramics. *J. Eur. Ceram. Soc.* **40**, 2936-2944 (2020).
148 Zhang, Y. & Zuo, R. Excellent energy-storage performances in La_2O_3 doped $(Na,K)NbO_3$-based lead-free relaxor ferroelectrics. *J. Eur. Ceram. Soc.* **40**, 5466-5474 (2020).
149 Zhang, M., Yang, H., Yu, Y. & Lin, Y. Energy storage performance of $K_{0.5}Na_{0.5}NbO_3$-based ceramics modified by $Bi(Zn_{2/3}(Nb_{0.85}Ta_{0.15})_{1/3})O_3$. *Chem. Eng. J.* **425**, 131465 (2021).
150 Xing, J. *et al.* Realizing high comprehensive energy storage and ultrahigh hardness in lead-free ceramics. *ACS Appl. Mater. Interfaces* **13**, 28472-28483 (2021).
151 Yang, Z. *et al.* A new family of sodium niobate-based dielectrics for electrical energy storage applications. *J. Eur. Ceram. Soc.* **39**, 2899-2907 (2019).
152 Fan, Y., Zhou, Z., Liang, R. & Dong, X. Designing novel lead-free $NaNbO_3$-based ceramic with superior comprehensive energy storage and discharge properties for dielectric capacitor applications via relaxor strategy. *J. Eur. Ceram. Soc.* **39**, 4770-4777 (2019).
153 Liu, Z., Lu, J., Mao, Y., Ren, P. & Fan, H. Energy storage properties of $NaNbO_3$-$CaZrO_3$ ceramics with coexistence of ferroelectric and antiferroelectric phases. *J. Eur. Ceram. Soc.* **38**, 4939-4945 (2018).
154 Pang, F. *et al.* Ultrahigh energy storage characteristics of sodium niobate-based ceramics by introducing a local random field. *ACS Sustainable Chem. Eng.* **8**, 14985-14995 (2020).
155 Qu, N., Du, H. & Hao, X. A new strategy to realize high comprehensive energy storage properties in lead-free bulk ceramics. *J. Mater. Chem. C* **7**, 7993-8002 (2019).
156 Shi, J. *et al.* Realizing ultrahigh recoverable energy density and superior charge–discharge performance in $NaNbO_3$-based lead-free ceramics via a local random field strategy. *J. Mater. Chem. C* **8**, 3784-3794 (2020).
157 Shi, J. *et al.* Superior thermal and frequency stability and decent fatigue endurance of high energy storage properties in $NaNbO_3$-based lead-free ceramics. *Ceram. Int.* **46**, 25731-25737 (2020).
158 Shi, R. *et al.* A novel lead-free $NaNbO_3$–$Bi(Zn_{0.5}Ti_{0.5})O_3$ ceramics system for energy storage application with excellent stability. *J. Alloys Compd.* **815**, 152356 (2020).
159 Yang, L., Kong, X., Cheng, Z. & Zhang, S. Enhanced energy storage performance of sodium niobate-based relaxor dielectrics by a ramp-to-spike sintering profile. *ACS Appl. Mater. Interfaces* **12**, 32834-32841 (2020).
160 Yang, Z. *et al.* Realizing high comprehensive energy storage performance in lead-free bulk ceramics via designing an unmatched temperature range. *J. Mater. Chem. A* **7**, 27256-27266 (2019).
161 Ye, J. *et al.* Excellent comprehensive energy storage properties of novel lead-free $NaNbO_3$-based ceramics for dielectric capacitor applications. *J. Mater. Chem. C* **7**, 5639-5645 (2019).
162 Zhou, M., Liang, R., Zhou, Z. & Dong, X. Superior energy storage properties and excellent stability of novel NaNbO 3-based lead-free ceramics with A-site vacancy obtained via a Bi_2O_3 substitution strategy. *J. Mater. Chem. A* **6**, 17896-17904 (2018).
163 Zhou, M., Liang, R., Zhou, Z., Yan, S. & Dong, X. Novel sodium niobate-based lead-free ceramics as new environment-friendly energy storage materials with high energy density, high power density, and excellent stability. *ACS Sustainable Chem. Eng.* **6**, 12755-12765 (2018).
164 Qi, H. *et al.* Ultrahigh energy-storage density in $NaNbO_3$-based lead-free relaxor antiferroelectric ceramics with nanoscale domains. *Adv. Funct. Mater.* **29**, 1903877 (2019).

165 Chen, J., Qi, H. & Zuo, R. Realizing stable relaxor antiferroelectric and superior energy storage properties in $(Na_{1-x/2}La_{x/2})(Nb_{1-x}Ti_x)O_3$ lead-free ceramics through A/B-site complex substitution. *ACS Appl. Mater. Interfaces* **12**, 32871-32879 (2020).

166 Tian, A., Zuo, R., Qi, H. & Shi, M. Large energy-storage density in transition-metal oxide modified $NaNbO_3$–$Bi(Mg_{0.5}Ti_{0.5})O_3$ lead-free ceramics through regulating the antiferroelectric phase structure. *J. Mater. Chem. A* **8**, 8352-8359 (2020).

167 Xie, A., Qi, H. & Zuo, R. Achieving remarkable amplification of energy-storage density in two-step sintered $NaNbO_3$–$SrTiO_3$ antiferroelectric capacitors through dual adjustment of local heterogeneity and grain scale. *ACS Appl. Mater. Interfaces* **12**, 19467-19475 (2020).

168 Xie, A. *et al.* $NaNbO_3$-$(Bi_{0.5}Li_{0.5})TiO_3$ lead-free relaxor ferroelectric capacitors with superior energy-storage performances via multiple synergistic design. *Adv. Energy Mater.* **11**, 2101378 (2021).

169 Chen, H. *et al.* Achieving ultrahigh energy storage density in $NaNbO_3$–$Bi(Ni_{0.5}Zr_{0.5})O_3$ solid solution by enhancing the breakdown electric field. *Ceram. Int.* **46**, 28407-28413 (2020).

170 Dong, X. *et al.* High energy storage density and power density achieved simultaneously in $NaNbO_3$-based lead-free ceramics via antiferroelectricity enhancement. *J. Materiomics* **7**, 629-639 (2021).

171 Dong, X. *et al.* High energy storage and ultrafast discharge in $NaNbO_3$-based lead-free dielectric capacitors via a relaxor strategy. *Ceram. Int.* **47**, 3079-3088 (2021).

172 Jiang, J. *et al.* Enhanced energy storage properties of lead-free $NaNbO_3$-based ceramics via A/B-site substitution. *Chem. Eng. J.* **422**, 130130 (2021).

173 Sun, C. *et al.* Simultaneously with large energy density and high efficiency achieved in $NaNbO_3$-based relaxor ferroelectric ceramics. *J. Eur. Ceram. Soc.* **41**, 1891-1903 (2021).

174 Chen, H. *et al.* Enhanced thermal and frequency stability and decent fatigue endurance in lead-free $NaNbO_3$-based ceramics with high energy storage density and efficiency. *J. Materiomics* **8**, 489-497 (2022).

175 Yan, F., Yang, H., Lin, Y. & Wang, T. Dielectric and ferroelectric properties of $SrTiO_3$–$Bi_{0.5}Na_{0.5}TiO_3$–$BaAl_{0.5}Nb_{0.5}O_3$ lead-free ceramics for high-energy-storage applications. *Inorg. Chem.* **56**, 13510-13516 (2017).

176 Wang, W. *et al.* Enhanced energy storage density and high efficiency of lead-free $Ca_{1-x}Sr_xTi_{1-y}Zr_yO_3$ linear dielectric ceramics. *J. Eur. Ceram. Soc.* **39**, 5236-5242 (2019).

177 Zhang, L. *et al.* Enhanced energy storage performance in Sn doped $Sr_{0.6}(Na_{0.5}Bi_{0.5})_{0.4}TiO_3$ lead-free relaxor ferroelectric ceramics. *J. Eur. Ceram. Soc.* **39**, 3057-3063 (2019).

178 Pan, W. *et al.* High breakdown strength and energy storage performance in (Nb, Zn) modified $SrTiO_3$ ceramics via synergy manipulation. *J. Mater. Chem. C* **8**, 2019-2027 (2020).

179 Cui, C., Pu, Y. & Shi, R. High-energy storage performance in lead-free $(0.8\text{-}x)SrTiO_3$-$0.2Na_{0.5}Bi_{0.5}TiO_3$-$xBaTiO_3$ relaxor ferroelectric ceramics. *J. Alloys Compd.* **740**, 1180-1187 (2018).

180 Yang, H., Yan, F., Lin, Y. & Wang, T. Improvement of dielectric and energy storage properties in $SrTiO_3$-based lead-free ceramics. *J. Alloys Compd.* **728**, 780-787 (2017).

181 Cui, C. *et al.* Structure, dielectric and relaxor properties in lead-free ST-NBT ceramics for high energy storage applications. *J. Alloys Compd.* **711**, 319-326 (2017).

182 Kong, X., Yang, L., Cheng, Z. & Zhang, S. Ultrahigh energy storage properties in $(Sr_{0.7}Bi_{0.2})TiO_3$-$Bi(Mg_{0.5}Zr_{0.5})O_3$ lead-free ceramics and potential for high-temperature capacitors. *Materials* **13**, 180 (2020).

183 Cui, C. & Pu, Y. Improvement of energy storage density with trace amounts of ZrO_2 additives fabricated by wet-chemical method. *J. Alloys Compd.* **747**, 495-504 (2018).

184 Yang, H., Yan, F., Lin, Y. & Wang, T. Enhanced recoverable energy storage density and high efficiency of $SrTiO_3$-based lead-free ceramics. *Appl. Phys. Lett.* **111**, 253903 (2017).

185 Yang, H., Yan, F., Lin, Y. & Wang, T. Novel strontium titanate-based lead-free ceramics for high-energy storage applications. *ACS Sustainable Chem. Eng.* **5**, 10215-10222 (2017).

186 Guo, X. *et al.* Ultrahigh energy storage performance and fast charge-discharge capability in Dy-modified $SrTiO_3$ linear ceramics with high optical transmissivity by defect and interface engineering. *Ceram. Int.* **46**, 21719-21727 (2020).

187 Li, J. F., Wang, S., Wakabayashi, K., Esashi, M. & Watanabe, R. Properties of modified lead zirconate titanate ceramics prepared at low temperature (800 °C) by hot isostatic pressing. *J. Am. Ceram. Soc.* **83**, 955-957 (2000).

188 Vittayakorn, N., Bongkarn, T. & Rujijanagul, G. Phase transition, mechanical, dielectric and piezoelectric properties of perovskite $(Pb_{1-x}Ba_x)ZrO_3$ ceramics. *Physica B: Condensed Matter* **387**, 415-420 (2007).

189 James, A., Rao, B. C., Pathak, M., Kamat, S. & Subrahmanyam, J. The effect of high energy mechanochemical processing on the microstructure, piezoelectric, ferroelectric and mechanical properties of PLZT ceramics. *Nanotechnology* **19**, 195201 (2008).

190 Li, Z., Sun, H., Liu, X., Sui, H. & Guo, H. High performance lead-free $Na_{0.5}K_{0.5}NbO_3$ piezoelectric ceramics obtained via oscillatory hot-pressing. *Ceram. Int.* **46**, 11617-11621 (2020).

191 Panteny, S., Bowen, C. & Stevens, R. Characterisation of barium titanate-silver composites, part I: Microstructure and mechanical properties. *J. Mater. Sci.* **41**, 3837-3843 (2006).

192 Karthik, T. & Asthana, S. Enhanced mechanical and ferroelectric properties through grain size refinement in site specific substituted lead free $Na_{0.5-x}K_xBi_{0.5}TiO_3$ (x=0–0.10) ceramics. *Mater. Lett.* **190**, 273-275 (2017).

193 Banerjee, K., Alvi, S. B., Rengan, A. K. & Asthana, S. Investigation on the discharge energy storage density of the Rb substituted $Na_{0.5}Bi_{0.5}TiO_3$ relaxor ferroelectric and its suitability for the orthopedic application. *J. Am. Ceram. Soc.* **102**, 6802-6816 (2019).

194 Jaita, P., Lertcumfu, N. & Rujijanagul, G. Temperature dependence on ferroelectric, energy storage density, and electric field-induced strain response of lead-free $Bi_{0.485}(Na_{0.388}K_{0.097})Ba_{0.021}Sr_{0.009}TiO_3$ ceramics. *Integr. Ferroelectr.* **201**, 142-154 (2019).

195 Guo, B. *et al.* Energy storage performance of $Na_{0.5}Bi_{0.5}TiO_3$ based lead-free ferroelectric ceramics prepared via non-uniform phase structure modification and rolling process. *Chem. Eng. J.*, 130475 (2021).

196 Kang, R. *et al.* Domain engineered lead-free ceramics with large energy storage density and ultra-high efficiency under low electric fields. *ACS Appl. Mater. Interfaces* **13**, 25143-25152 (2021).

197 Yan, F. *et al.* Superior energy storage properties and excellent stability achieved in environment-friendly ferroelectrics via composition design strategy. *Nano Energy* **75**, 105012 (2020).

198 Ye, H. *et al.* Significantly improvement of comprehensive energy storage performances with lead-free relaxor ferroelectric ceramics for high-temperature capacitors applications. *Acta Mater.* **203**, 116484 (2021).

199 Xing, J. *et al.* Realizing high comprehensive energy storage and ultrahigh hardness in lead-free ceramics. *ACS Appl. Mater. Interfaces* **13**, 24, 28472-28483 (2021).

200 Zhao, P. *et al.* Structure, dielectric and relaxor properties of $Sr_{0.7}Bi_{0.2}TiO_3K_{0.5}Bi_{0.5}TiO_3$ lead-free ceramics for energy storage applications. *J. Materiomics* **7**, 195-207 (2021).
